# Water-Soluble Melanoidin Pigment as a New Antioxidant Component of Fermented Willowherb Leaves (*Epilobium angustifolium*)

**DOI:** 10.3390/antiox10081300

**Published:** 2021-08-18

**Authors:** Daniil N. Olennikov, Christina S. Kirillina, Nadezhda K. Chirikova

**Affiliations:** 1 Laboratory of Medical and Biological Research, Institute of General and Experimental Biology, Siberian Division, Russian Academy of Science, 670047 Ulan-Ude, Russia; 2 Department of Biology, Institute of Natural Sciences, North-Eastern Federal University, 677027 Yakutsk, Russia; kristinakirillina22@gmail.com (C.S.K.); hofnung@mail.ru (N.K.C.)

**Keywords:** *Epilobium angustifolium*, onagraceae, fermentation, melanoidin, liquid chromatography-mass spectrometry, antioxidant activity, simulated digestion

## Abstract

Willowherb (*Epilobium angustifolium* L., family Onagraceae) is a well-known food and medicinal plant used after fermentation as a source of beverages with high antioxidant potential. Despite this long history of use, only a few papers have described the chemical profile and bioactivity of fermented willowherb tea in general. To understand the basic metabolic differences of non-fermented and fermented *E. angustifolium* leaves, we used general chemical analysis, high-performance liquid chromatography with photodiode array detection and electrospray ionization triple quadrupole mass spectrometric detection assay, and an isolation technique. As a result, the content of 14 chemical groups of compounds was compared in the two plant materials; 59 compounds were detected, including 36 new metabolites; and a new water-soluble phenolic polymer of melanoidin nature was isolated and characterized. The fundamental chemical shifts in fermented *E. angustifolium* leaves relate mainly to the decrease of ellagitannin content, while there is an increase of melanoidin percentage and saving of the antioxidant potential, despite the significant changes detected. The strong antioxidative properties of the new melanoidin were revealed in a series of in vitro bioassays, and a simulated gastrointestinal and colonic digestion model demonstrated the stability of melanoidin and its antioxidant activity. Finally, we concluded that the new melanoidin is a basic antioxidant of the fermented leaves of *E. angustifolium,* and it can be recommended for additional study as a promising food and medicinal antioxidant agent.

## 1. Introduction

Fermentation, as a biotechnological process, is widely used in the food industry to modify various organoleptic and physicochemical properties of plant materials, including the colour, odour, taste, and extractability [1]. Recently, there has been increased scientific interest in fermented products from medical science, which has led to the creation of a large number of functional products with a certain biological activity [2,3]. The main advantage of functional products is a mild correction of a lack of vitamins, minerals, antioxidants, and other nutraceuticals without fear of excessive consumption [4,5,6]. Widely used functional products are functional beverages, namely herbal teas, which have pleasant organoleptic properties and a positive biological effect [7]. The application of fermentation to create plant-based beverages is the most commonly used process.

Among the functional drinks obtained from fermented plants, the most famous are those using *Camellia sinensis* (L.) Kuntze (Theaceae) to create teas with a wide range of fermentation [8,9]. The most fermented product, black tea, is the most popular functional drink along with green tea. The chemical feature of such beverages is the presence of low-molecular-weight metabolites of the unfermented plant and a specific group of phenolic polymers, melanoidins (melanins), which give a dark colour to the *C. sinensis* decoction [10]. These polymers are not indifferent and have biological activity, causing antioxidant, metal chelating, and anti-inflammatory effects of black tea decoctions [11]. A melanoidin similar in structure and biological activity was also isolated from *Orthosiphon aristatus* (Blume) Miq. (Java tea; Lamiaceae) [12].

Along with those made from the aforementioned plants, interest in other fermented beverages has been growing recently, in particular regarding those obtained from the plant *Epilobium angustifolium* L. (willowherb, rosebay, fireweed; *Chamaenerion angustifolium* (L.) Scop., *Chamerion angustifolium* (L.) Holub) [13,14,15]. This drink, which has a taste similar to that of black tea and has intense dark pigmentation, is considered a new product for medicine but has a rich history of use in Russia and some European countries under the name Russian tea or Koporye tea (koporskii chai; derived from the Old Russian word ‘*κonpъ*’ meaning ‘fragrant plant’ or Koporye village name).

There is no reliable information on the invention of Koporye tea, but the most probable fact is the accidental fermentation of the fresh herb of *E. angustifolium*, which was used unprocessed to brew tea [16]. Information about the use of Koporye tea in Russia dates back to the XI–XII centuries; however, the first official document that mentions this drink is the Regulation of the Council of Ministers ‘On the prohibition of counterfeiting Koporye tea under the guise of Chinese tea’ dated June 6, 1816 [17]. At the time, the fermented *E. angustifolium* herb was applied as a local tea drink as well as a cheap surrogate and counterfeit for black tea imported from China [18]. More than two centuries later, there was renewed interest in Koporye tea due to its well-known tonic properties.

The parent plant species for Koporye tea is *E. angustifolium*, a well-studied plant that contains many groups of phenolic compounds with different structures, as well as terpenoids and essential oils [19,20,21,22]. Pharmacological studies of *E. angustifolium* extracts have shown a wide range of biological activity, including antioxidant, anti-inflammatory, antitumour, and enzyme inhibiting. In addition to the good scientific knowledge of *E. angustifolium*, studies of fermented willowherb are rare.

It is known that *E. angustifolium* leaves (herb) after aerobic and anaerobic fermentation contain organic acids, carotenoids, ellagitannins, flavonoids, hydroxycinnamates, benzoic acids, and essential oils, and the total extracts have antioxidant activity [13,14,15]. Despite the known specificity of changes in the chemical composition of *E. angustifolium* after fermentation, such as the loss or accumulation of low-molecular-weight compounds, the total number of characterized compounds does not exceed 20 [19,20,21,22]. While scientists have given the issue some attention, it is not sufficient, and more specific metabolic data need to be obtained from liquid chromatography-mass spectrometric analysis.

The unstudied question of the nature of the pigment that causes the browning of *E. angustifolium* leaves after fermentation deserves special attention. The most likely explanation for this phenomenon is the formation of melanoidins, similar to those in the well-known fermented black tea [10] or Java tea drinks [12]. This suggests the presence of various types of biological activity in the pigment [11]. To close these gaps, we realized a comprehensive comparative study of *E. angustifolium* leaves before and after fermentation including metabolomic characterization by high-performance liquid chromatography with photodiode array detection and an electrospray ionization triple quadrupole mass spectrometric detection assay (HPLC-PDA–ESI-tQ-MS/MS).

In this work, for the first time, the melanoidin from fermented leaves of *E. angustifolium* was isolated and its physicochemical properties were studied, as well as its antioxidant activity using in vitro methods without digestion and under simulated gastrointestinal and colonic digestion. Studies have shown that the fermentation of *E. angustifolium* is a much more complex process than previously described, leading to the formation of a new functional product.

## 2. Materials and Methods

### 2.1. Plant Material and Chemicals

A sample of *Epilobium angustifolium* fresh leaves (25 kg) was collected in the Republic of Sakha Yakutia (Chulman, Aldanskii Ulus, 25.VI.2020, 57°00′37″ N, 124°49′02″ E, 960 m a.s.l.; voucher No YA/ONA-0620/53-269). The species was authenticated by Dr. N.I. Kashchenko (IGEB SB RAS, Ulan-Ude, Russia). The plant material was divided into two parts, and one part was dried in the ventilated heat oven at 40 °C within 7–10 days and stored at 3–4 °C before analysis. The second part of fresh plant material was fermermented as described in Section 2.2. The reference compounds were purchased from Biopurity Phytochemicals Ltd. (Chengdu, China), ChemFaces (Wuhan, Hubei, China), Extrasynthese (Lyon, France), MCE Med Chem Express (Monmouth, NJ, USA), and Sigma-Aldrich (St. Louis, MO, USA) (Table S1).

### 2.2. Epilobium Angustifolium Leaves Fermentation

The fresh leaves of *E. angustifolium* (10 kg; moisture content 84.7%) without pretreatment were loaded into the climate chamber KMF720 (Binder GmbH, Tuttlingen, Germany; 28 °C, humidity 90%) as a thin layer and thermostated 24 h. Then, the partially wilted plant material was loaded into the 3-Dimensional Blender GH-60 (Harbin, Heilongjiang, China) and rotated at 10 rpm and 25 °C for 90 min. The curled material was returned to the climate chamber KMF720 and thermostated again at 35 °C and 95% humidity with aeration for 96 h. The final drying of the *E. angustifolium* fermented leaves was performed in the Convectional Oven Climcontrol SHS 30/250-3000-P Standard (Mir Oborudovania Ltd., St Petersburg, Russia) at 95 °C with constant air convection for 3 h. The yield of dry fermented leaves of *E. angustifolium* was 1.42 kg (14.2% from the fresh plant weight; moisture content 4.6%).

### 2.3. Antioxidant Activity Assays

The values of the hydrophilic oxygen radical absorbance capacity (H-ORAC) and lipophilic oxygen radical absorbance capacity (L-ORAC) of *E. angustifolium* leaves before and after fermentation and black tea of Lipton^®^ brand were determined using a fluorimetric microplate assay [23]. The H-ORAC and L-ORAC values were found after the building of the regression between trolox concentration (μM) and the net area under the fluorescence decay curve. The trolox diluted by 0.075 M phosphate buffer at the range 6.25–50.00 μM. Data were expressed as trolox equivalents (μM) per 1 g of dry plant. The total antioxidant capacity (TAC) was calculated as a sum of the H-ORAC and L-ORAC values. All the analyses were carried out six times, and the data are expressed as the mean value ± standard deviation (S.D.).

The antioxidant potential of *E. angustifolium* melanoidin, oenothein B and trolox was studied using the known in vitro microplate spectrophotometric assays: scavenging activity against 2,2-diphenyl-1-picrylhydrazyl radicals (DPPH^•^) [24], 2,2′-azino-bis(3-ethylbenzothiazoline-6-sulfonic acid) cation radicals (ABTS^•^^+^) [25], *N*,*N*-dimethyl-*p*-phenylenediamine radicals (DMPD^•+^) [26], superoxide anion radicals (O_2_^•^^−^) [27], hydroxyl radicals (^•^OH) [27], and chloride radicals (Cl^•^) [28]. The nitric oxide (II) scavenging potential was studied in a spectrophotometric assay with sodium nitroprusside as a source of NO [29]. The chelating activity of ferrous (II) ion was determined by spectrophotometric assay [30]. The results of following assays DPPH^•^, ABTS^•+^, DMPD^•+^, O_2_^•−^, ^•^OH, and NO were measured as the half maximal inhibitory concentration or IC_50_ calculated graphically using ‘concentration (μg/mL or mg/mL)–antioxidant activity (%)’ correlations. All the analyses were carried out five times, and the data are expressed as the mean value ± standard deviation (S.D.).

### 2.4. Chemical Composition Analysis

The total content of the water- and methanol-soluble extracts in *E. angustifolium* leaves was determined using a gravimetric assay [31]. The total lipids were extracted by a chloroform/methanol/water mixture (1:2:0.8) followed the known Bligh–Dyer quantitative assay [32]. Spectrophotometric analysis was performed using a UV-Vis spectrophotometer SF-2000 (OKB Spectr, Saint Petersburg, Russia) for the quantitative determination of photosynthetic pigments (carotenoids and chlorophylls) [25], polysaccharides (as mg/g glucose equivalents) [33], phenolics (as mg/g gallic acid equivalents) [34], and flavonoids (as mg/g miquelianin equivalents) [35].

A Spectrophotometric Plate Reader Multiskan Go (Thermo Fisher Scientific Inc., Waltham, MA, USA) was used for the quantitative analysis of proteins (as mg/g BSA equivalents; Protein Assay Kit II; BioRad Laboratories Inc., Hercules, CA, USA; cat. No 5000002), free amino acids (as a sum of L- and D-amino acids determined by L-Amino Acid Assay Kit (Abcam, Cambridge, UK; cat. No ab65347) and D-Amino Acid Assay Kit (Abcam; cat. No ab239721), respectively), monosaccharides (as mg/g glucose equivalents; d-Fructose/d-Glucose Assay Kit, Megazyme Ltd., Gatton, Australia; cat. No K-FRGLQR), and disaccharides (as mg/g sucrose equivalents; EnzyChrom™ Sucrose Assay Kit, BioAssay Systems, Hayward, CA, USA; cat. No ESUC-100).

Titratable acids were quantified using a titration assay [36] with 0.1 M sodium hydroxide as the titrant and expressed as mg/g malic acid equivalents. To measure the melanin content, 100  mL of bidistilled water was added to 1 g of powdered plant and heated at 90 °C for 60  min. The water extract was filtered, acidified with 1 mL of concentrated hydrochloric acid, and the pigmented precipitate was collected after centrifugation at 6000× *g* for 30 min. Finally, the precipitate was washed with 5% acetic acid and dried in vacuo. The melanin yield was expressed as mg per 1 g of dry plant. All the analyses were carried out five times, and the data are expressed as the mean value ± standard deviation (S.D.).

### 2.5. High-Performance Liquid Chromatography with Photodiode Array Detection and Electrospray Ionization Triple Quadrupole Mass Spectrometric Detection (HPLC-PDA-ESI-tQ-MS)

High-performance liquid chromatography with photodiode array detection and electrospray ionization triple quadrupole mass spectrometric detection (HPLC-PDA-ESI-tQ-MS) was used for the quantitative analysis of metabolites of *E. angustifolium* leaves before and after fermentation. Liquid chromatograph LC-20 Prominence coupled with photodiode array detector SPD-M30A (wavelength range 200–600 nm), triple-quadrupole mass spectrometer LCMS 8050 (all Shimadzu, Columbia, MD, USA) and GLC Mastro column (2.1 × 150 mm, 3 μm; Shimadzu, Kyoto, Japan) used two-eluent gradient elution for the successful separation of compounds.

The eluents A and B were 0.5% HCOOH in water and 0.5% HCOOH in MeCN, respectively, and the following gradient program was used for the separation: 0–3 min 5%, 3–8 min 5–9%, 8–10 min 9–16%, 10–18 min 16–33%, 18–24 min 33–38%, 24–27 min 38–40%, 27–35 min 40–50%, 35–40 min 50–72%, and 40–50 min 72–5%. The injection volume and the flow rate were 1 μL and 150 μL/min, respectively. The UV-Vis spectral range was 200–600 nm. The negative ESI mode was used for the mass spectrometric detection, and the following temperatures were used for the ESI interface: 300 °C, desolvation line 250 °C and heat block 400 °C.

Various flow values were used for the nebulizing gas (N_2_) 3 L/min, heating gas (air) 10 L/min, and collision-induced dissociation gas (Ar) 0.3 mL/min. The source voltage of registered mass spectra was 3 kV, and the collision energy was −10–35 eV for the scanning range of *m*/*z* 80–1900. The managing of the LC-MS system was realized by LabSolution’s workstation software (Columbia, MD, USA) equipped with the inner LC-MS library.

The final identification of metabolites was performed after an integrated analysis of the retention time, ultraviolet, and mass spectra in comparison with the reference standards and literature data. The dried plant samples of *E. angustifolium* leaves before and after fermentation (200 mg) were extracted with 50% methanol (2 mL) four times with sonication (ultrasonic bath, 20 min, 40 °C, ultrasound power 100 W, frequency 35 kHz). The extracts were filtered through 0.22 μm syringe filters into the measuring flask (10 mL), and the final volume was reached 10 mL with 50% methanol. The extracts were stored at 0 °C before the HPLC-PDA-ESI-tQ-MS analysis.

### 2.6. Metabolite Quantification

For the quantification of metabolites in *E. angustifolium* leaves before and after fermentation the known HPLC-PDA-ESI-tQ-MS conditions used (Section 2.5) and full scan MS peak area used for calculation. Fifty-nine compounds were quantified, and 31 reference standards were used for the building of calibration curves (Table S1). The selected reference standard was carefully weighed (10 mg), dissolved in the methanol-DMSO mixture (1:1) in volumetric flasks (10 mL) to prepare the stock solution 1000 µg/mL.

A series of the calibration solution 1–100 µg/m was used to create the calibration curve based on the ‘concentration–mass spectrometric peak area’ correlation. Selected validation criteria, including the correlation coefficients (r^2^), standard deviation (S_YX_), limits of detection (LOD), limits of quantification (LOQ), and linear ranges, were found using the known methodology [37] (Table S2). Five-fold repetition was used for all quantitative analyses, and the results are expressed as the mean value ± standard deviation (S.D.).

### 2.7. Melanoidin Isolation

To isolate *E. angustifolium* melanoidin pigment, fermented leaves (500 g) were powdered and extracted with chloroform (Soxhlet Extractor, 65 °C, 48 h) and ethyl acetate (sonication, 40 °C, ultrasound power 100 W, frequency 35 kHz, 90 min) to remove the lipids and low-molecular phenolics. The pre-treated dry plant powder was extracted two times with sonification (ultrasound power 150 W, frequency 35 kHz) with distilled water at 85 °C using a solid: liquid ratio of 1:15 (*w*/*v*). The resultant mixture was filtered, the water extract was concentrated under vacuum at 40 °C to a final volume of 1 L and the residue was centrifuged at 6000× *g* for 30 min.

The resulting solution was acidified with 25% HCl to a final concentration of 1% and stored for 6 h at 10 °C. The brown precipitate was centrifuged at 10,000× *g* for 30 min, washed with 1% HCl, and dissolved in 250 mL water. Three volumes of acetone were added to precipitate polysaccharide and protein impurity, and the mixture was centrifuged at 10,000× *g* for 20 min. The supernatant was concentrated under a vacuum at 40 °C to dry the residue, which was dissolved in 200 mL of bidistilled water and dialyzed 72 h in dialyzing tubes with a cut-off limit of 2 kDa (Sigma-Aldrich, cat. No D7884). The undialysed fraction was dried by lyophilization to give 62.5 g of crude melanoidin (yield 12.5% of dry plant weight).

The powder of crude melanoidin was suspended in 20% HCl (1:5) and stored for five days at 5 °C. The powder was centrifuged at 6000× *g* (30 min), washed with 1% HCl, dissolved in bidistilled water, and dialyzed again. The undialysed part was dried by lyophilization and dissolved in 100 mL of bidistilled water, and the solution was passed through a Sephadex G-150 column (110 × 5 cm) coupled to an SF-2000 UV-Vis flow detector (OKB Spectr, Saint Petersburg, Russia); 7-mL fractions were collected using bidistilled water as the eluent. The fractions No. 80–105 were combined and dried by lyophilization, giving fractions containing melanoidin EAM. The yield of EAM was 8.75 g (14% of crude melanoidin weight).

### 2.8. Melanoidin Analysis

#### 2.8.1. High-Performance Gel Permeation Chromatography with Photodiode Array Detection

The molecular weight analysis of melanoidin EAM was determined using high-performance gel permeation chromatography with photodiode array detection (HPGPC-PDA) at liquid chromatograph LC-20 Prominence coupled with photodiode array detector SPD-M30A (wavelength 270 nm; all Shimadzu) and TSK-Gel G4000 SWXL column (7.8 × 300 mm; Shimadzu, Tosoh Bioscience LLC, Tokyo, Japan) at the column temperature of 35 °C. Isocratic elution with 20 mM KH_2_PO_4_ was used for the separation. The injection volume was 5 μL, and the flow rate was 500 μL/min. To calibrate the column, the dextrans with 10, 400, and 400 kDa were used (all Sigma-Aldrich).

#### 2.8.2. Functional Group Quantification

The contents of carboxylic groups and phenolic hydroxyl groups were measured by a pH-meter 410 (Akvilon, Russia) using potentiometric assays [38]. The pyrocatechol group content was determined using the FeSO_4_/tartrate method with pyrocatechol as a standard [39].

#### 2.8.3. Elemental Composition

Elemental analysis (CHNS/O) was performed in a 2400 Series II elemental analyser (Perkin Elmer, Waltham, MA, USA).

#### 2.8.4. Spectroscopy in Ultraviolet and Visible Region (UV-Vis)

The UV-Vis spectra in the spectral range 190–1000 nm were studied using a UV-Vis spectrophotometer SF-2000 (OKB Spectr) using a quartz cell (10 mm) and series of melanoidin EAM solutions in bidistilled water (3.9–1000 μg/mL) with bistilled water was as a blank. The optical density data was used to build a graph of the optical density logarithm (common logarithm) against the wavelength followed by the determination of the linear regression equation and the value of the slope, which is a logarithmic slope of absorbance.

The value of the chromatic coefficient E_465_/E_665_ was calculated as a ratio of the optical densities at 465 and 665 nm for melanoidin EAM solution (31.3 μg/mL) in bidistilled water. To find the color value E^1%^_1 cm_ of melanoidin, a series of EAM solutions (3.9–1000 μg/mL) was used to creation of the graph of the optical density of EAM versus concentration and the linear regression was used to find the regression equation. Finally, the optical density of a 1% EAM solution was found as a color value (E^1%^_1 cm_). Three-fold repetition was used for all spectral studies.

#### 2.8.5. Fourier-Transform Infrared Spectroscopy (FTIR)

Spectra were recorded using a Fourier-Transform Infrared Spectrometer FT-801 (Simex, Novosibirsk, Russia; frequency 4000–600 cm^−1^) with 140 scans and 2 cm^−1^ resolution. The substances were measured with KBr tablet (spectroscopic grade) powder in a ratio of 1:100 (substance: KBr).

#### 2.8.6. Nuclear Magnetic Resonance (NMR) Spectroscopy

^13^C NMR spectrum was recorded in a NMR spectrophotometer VXR 500S (Varian, Palo Alto, CA, USA) at a working frequency of 125.7 MHz. The spectrum was obtained for a 1% solution in 5% NaOD.

#### 2.8.7. Alkaline Destruction

A weighed sample of melanoidin EAM (1 g) was heated to 250 °C in a porcelain crucible with 100 mg of finely ground KOH for 5 min. After cooling, the mixture was dissolved in 20 mL of water, the resultant solution was neutralized with 80% H_2_SO_4_ to pH 4–5, and then liquid-liquid extracted with ethyl acetate (5 mL × 5). The organic extracts were vacuum dried, dissolved in 1 mL of methanol, and analyzed using an HPLC-PDA-ESI-tQ-MS assay (Section 2.8.8).

#### 2.8.8. HPLC-PDA-ESI-tQ-MS Analysis of Alkaline Destruction Products

The known apparatus (Section 2.5) used for HPLC-PDA-ESI-tQ-MS analysis of melanoidin EAM alkaline destruction products with different eluents and gradient program. A two-eluent gradient elution was used for the separation: 0.5% HClO_4_ in water as the A eluent and 0.5% HClO_4_ in MeCN as the B eluent. The following gradient program was used: 0–7 min 5–45% B, 7–12 min 45–72% B, 12–15 min 72–84% B, and 15–25 min 84–5%. The injection volume was 1 μL, and the flow rate was 100 μL/min.

### 2.9. Plant Decoction Preparation

The samples of non-fermented and fermented leaves of *E. angustifolium* (1 g) were extracted with 100 mL of distilled water by heating on a Heater Plate USP 1M (Sorbfil, Krasnodar, Russia) with 10 min boiling. Before filtration, the water decoction was cooled (20 °C) and then filtered through a paper filter.

### 2.10. Simulated Gastrointestinal and Colonic Digestion

The previously described methods were used for the simulated gastrointestinal [23] and colonic digestion [40]. The samples of melanoidin EAM (100 mg), oenothein B (100 mg), and *E. angustifolium* leaf decoctions (10 mL) were incubated at 37 °C within 60 min in a shaking water bath (167 rpm) with 25 mL of simulated gastric fluid with pH 2.0 (gastric phase) [23] followed by the addition of 1 mL of bile solution and 4 mL simulated intestinal fluid with pH 7.0 [23] and 4 h stirring in the dialysis bag at 37 °C against 1000 mL of clear simulated intestinal fluid (no pancreatin) (intestinal phase).

An aliquot (5 mL) of the retentate (non-dialyzed part of intestinal phase sample), fecal slurries (donated by healthy volunteers [40]; 1 g in 5 mL of brain heart infusion) and brain heart infusion (15 mL) were mixed and anaerobically incubated at 37 °C for 48 h using BD GasPak^TM^ EZ anaerobe container system sachets (Franklin Lakes, NJ, USA) (colonic phase). An aliquot (100 μL) of reaction mixture before simulation and after the gastric, intestinal, and colonic phase were measured coulometrically to determine the trolox equivalent content in probe (Section 2.11).

### 2.11. Coulometric Analysis of Trolox Equivalent Content

The trolox equivalent content in the oenothein B and *E. angustifolium* melanoidin solutions and the *E. angustifolium* decoctions was found by a coulometric titration method with electrogenerated bromine radicals [28] using the potentiostat Expert-006 (Econics Expert Ltd., Moscow, Russia) with a four-electrode two-compartment electrochemical cell. All measurements were carried out three times.

### 2.12. Statistical Analysis

One-way analysis of variance was applied for the statistical analyses, and Duncan’s multiple range test was used to detect the significance of the mean difference. The values of the differences were statistically significant at *p* < 0.05. Advanced Grapher 2.2 (Alentum Software Inc., Ramat-Gan, Israel) was used for the linear regression analysis and building of calibration graphs.

## 3. Results and Discussion

### 3.1. Fermentation of Epilobium angustifolium: Antioxidant Potency and Chemical Profile of Non-Fermented and Fermented Leaves

**Figure 1:**
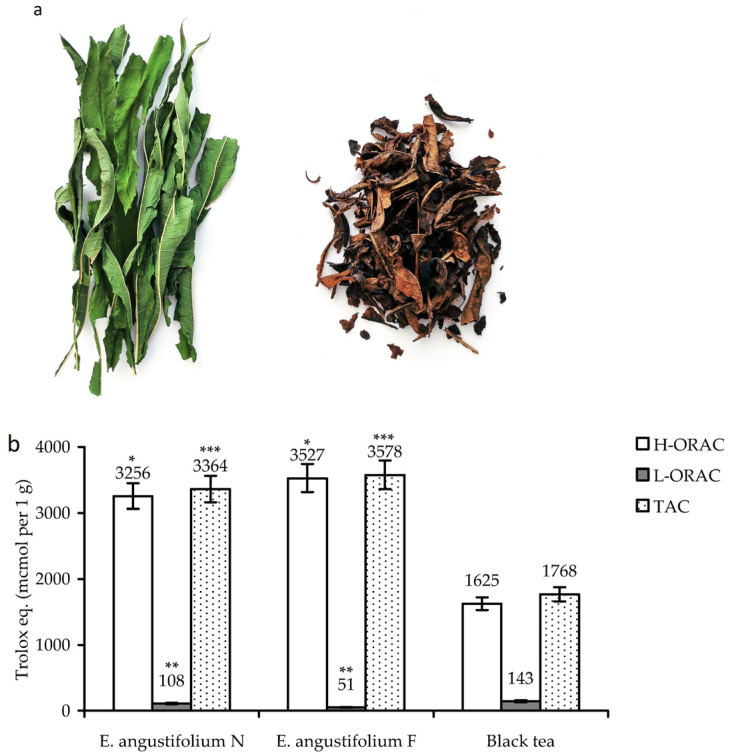
(**a**) Dried leaves of *Epilobium angustifolium* before (left) and after fermentation (right). (**b**). Hydrophilic (H-ORAC) and lipophilic (L-ORAC) antioxidant capacity and total antioxidant capacity (TAC; as Trolox equivalents, μmol per gram) of *E. angustifolium* leaves before (*E. angustifolium* N) and after fermentation (*E. angustifolium* F) in comparison with black tea (Lipton^®^ tea). The results show the mean ± S.D. of five experiments. * *p* < 0.01 vs. black tea H-ORAC group; ** *p* < 0.01 vs. black tea L-ORAC group; and *** *p* < 0.01 vs. black tea TAC group.

In this work, the traditional Russian method of processing leaves of *E. angustifolium* was used to obtain fermented material in a a four-stage process. As a result, the green leaves of *E. angustifolium* lost their natural shape and color and acquired a characteristic ‘tea’ color and a specific aromatic odor (Figure 1a).

Comparative analysis of the hydrophilic (H-ORAC)/lipophilic (L-ORAC) antioxidant capacity and total antioxidant capacity (TAC) of dried leaves of *E. angustifolium* before and after fermentation showed their similar potential (Figure 1b). The H-ORAC value of non-fermented and fermented leaves was 3256 and 3527 μmol/g, respectively, while the L-ORAC values were much lower, 108 and 51 μmol/g, respectively. The TAC parameter of non-fermented leaves of *E. angustifolium* (3364 μmol/g) was not considerably different from that of fermented leaves (3578 μmol/g), thus, indicating statistically insignificant differences in the antioxidant properties of the two plant materials.

The commercial black tea (Lipton^®^ tea) was less active, with a TAC value of 1768 μmol/g, which is the case for the majority of black tea brands [41]. This means that fermentation of *E. angustifolium* leaves does not affect the antioxidant properties of the plant material despite the serious external changes. Similar results were obtained in earlier studies of *E. angustifolium* in which fermentation did not lead to pronounced changes in the antioxidant properties [13,14,15].

To understand the chemical changes that happened in *E. angustifolium* leaves after fermentation, we quantified various compounds, such as the extractives, lipids, proteins, amino acids, carbohydrates, organic acids, and phenolics (Table 1). Preliminary analysis of the total extractives content in leaves of *E. angustifolium* before and after fermentation showed that the fermentation resulted in an increase of both water-soluble (28.42%→38.53%) and methanol-soluble extractives (41.20%→49.63%). The values for the total lipids and carotenoids did not change much; however there was a dramatic loss of chlorophyll, from 8.65 mg/g to trace.

The concentration of proteins and water-soluble polysaccharides decreased; however, the amino acid and mono-/disaccharide levels were much higher in fermented leaves as was the titratable acidity value. All this indicates that the fermentation had a weak impact on the lipophilic compounds (except the chlorophylls, which probably degraded) but proteins disintegrated into amino acids, and water-soluble polysaccharides gave a mixture of mono- and disaccharides. This is understandable because aerobic fermentation releases various enzymes in plant cells, contributing to the degradation of macromolecular compounds to simple components [1,2,3,4,5,6].

The changes in the total phenolic content were not significant (277.15 mg/g→252.11 mg/g); however, inside of this class of compounds, significant changes were observed (Table 1). The tannins were the most deeply affected compounds, with a 93% loss of content, in contrast to flavonoids, which were almost unchanged (52.73 mg/g→50.61 mg/g). The accumulation of a new component, melanoidin, missing in the original plant, caused the browning of plant material. The nature of the melanoidin from *E. angustifolium* fermented leaves is still unknown; therefore, it will be subsequently studied.

**Figure 2:**
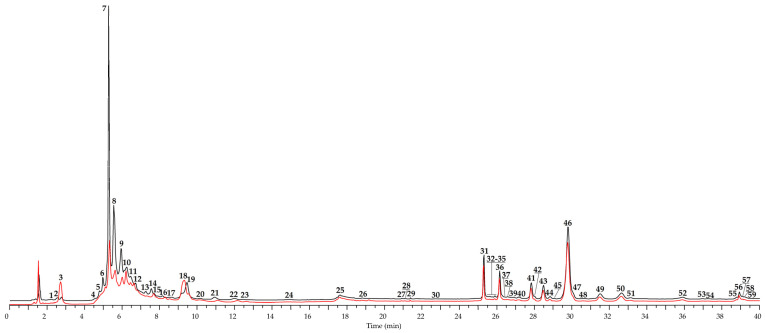
High-performance liquid chromatography with photodiode array detection (HPLC-PDA) chromatogram (270 nm) of *E. angustifolium* leaves before (black) and after fermentation (red). Compounds are numbered as listed in Table 2.

The changes of low-molecular-weight phenolics before and after the fermentation of *E. angustifolium* leaves were precisely analysed by high-performance liquid chromatography with photodiode array detection and electrospray ionization triple quadrupole mass spectrometric detection (HPLC-PDA–ESI-tQ-MS). Finally, 59 compounds were identified after comparison of their chromatographic behaviour, ultraviolet, and mass spectra with reference substances (28 compounds) and literature data [27,28,42,43,44,45,46,47,48,49,50,51,52,53,54] (Figure 2, Table 2). Only 23 of the compounds had been found in *E. angustifolium* previously [43,45,54]; 36 compounds were detected for the first time.

Various phenolics were found in *E. angustifolium* leaves, such as gallic acid glycosides, ellagitannins, ellagic acid glycosides, hydroxycinnamates, and flavonol glycosides. Gallic acid (**3**) has been reported previously in willowherb [43] but its hexosides, such as glucogallin (**1**) and isomer (**2**), have not.

Six known ellagitannins of relative nature, oenothein B (**7**), oenothein A (**8**), tetrameric/pentameric/hexameric/heptameric tellimagrandin I (**9**–**12**), were finely described in early studies of European samples of *E. angustifolium* [43,45,47]. In addition, unknown galloyl-hexahydroxydiphenoyl-di-*O*-hexosides **4** and **5** (*m*/*z* 795 [M − H]^−^) and galloyl-hexahydroxydiphenoyl-*O*-hexoside **6** (*m*/*z* 633 [M − H]^−^) were detected.

The large group of ellagic acid derivatives contained 16 compounds. Free ellagic acid (**18**) is a usual component of plants of the genus *Epilobium* [22], in contrast to ellagic acid *O*-hexosides, which were found in the genus for the first time. Comparison with reference standards allowed the identification of 1-*O*-ellagoyl-gentiobiose (amritoside, **14**) [55], 1,6′-di-*O*-ellagoyl-gentiobiose (granatoside A, **16**) [48], 1-*O*-ellagoyl-glucose (**17**) [48], and 1,6-di-*O*-ellagoyl-glucose (granatoside B, **19**) [48].

The remaining derivatives were di-ellagoyl-tri-*O*-hexose **15** (*m*/*z* 1071 [M − H]^−^), ellagic acid-tri-*O*-hexose-*O*-acetate **20** (*m*/*z* 829 [M − H]^−^), di-ellagoyl-tri-*O*-hexose-*O*-acetate **21** (*m*/*z* 1113 [M − H]^−^), and five *O*-hexosides of ellagic acid *O*-methyl esters (**22**–**26**), which gave deprotonated ions with *m*/*z* 477, 491, 761, 775, and 505. Two lipophilic esters of ellagic acid were found: 3-*O*-methyl ester **53** and 3,3′-di-*O*-methyl ester **54**.

Seven hydroxycinnamates were identified as 1- and 6-*O*-sinapoyl-glucose (**27**, **28**) [49], 1-*O*-caffeoyl-glucose (**29**) [49], 1-, 4-, and 5-*O*-caffeoylquinic acids (**30**, **31**, **36**) [50], and an unknown di-*O*-caffeoyl-hexose **32** (*m*/*z* 503 [M − H]^−^). Only caffeoylquinic acid **36** was a known willowherb compound [43].

The flavonoids of *E. angustifolium* were flavonol glycosides of kaempferol, quercetin, and myricetin series. Among the kaempferol glycosides, the known *E. angustifolium* flavonoids were 3-*O*-glucuronide **50** [43], 3-*O*-rhamnoside **52** (afzelin) [43], and 3-*O*-(6″-*O*-*p*-coumaroyl)-glucoside **57** (tiliroside) [53], as well as the unknown *O*-galloyl-*O*-hexoside **48** and *O*-acetyl-*O*-*p*-coumaroyl-*O*-hexoside **59**. Quercetin glycosides were the most diverse group of flavonoids, including 3-*O*-(6″-*O*-galloyl)-galactoside **43** [43], 3-*O*-glucuronide **46** (miquelianin) [43], 3-*O*-galactoside **47** (hyperoside) [43], 3-*O*-arabinoside **49** (avicularin) [43], and 3-*O*-rhamnoside **51** (quercitrin) [43] found in early studies of the *E. angustifolium* herb.

The presence of rutin (**45**), helichrysoside (**56**), and five unknown compounds (**34**, **35**, **38**, **40**, **58**) in *E. angustifolium* leaves was shown for the first time. Myricetin 3-*O*-galactoside **42** and 3-*O*-rhamnoside **44** (myricitrin) have previously been reported in *E. angustifolium* herb [43] as well as myricetin *O*-hexuronide **41** [45] and *O*-galloyl-*O*-hexoside **39 [43]**. The new flavonoids of *E. angustifolium* were myricetin di-*O*-galloyl-*O*-hexoside-*O*-hexuronide **33**, *O*-galloyl-*O*-hexoside-*O*-hexuronide **37**, and *O*-*p*-coumaroyl-*O*-hexoside **55**.

The information obtained by the HPLC-PDA–ESI-tQ-MS assay indicates that the chemodiversity of *E. angustifolium* of Asian origin is close to that of European samples [43,45,47,52,54] but with specific metabolic markers, namely ellagic acid glycosides, hydroxycinnamates, and acylated flavonol glycosides. A possible reason for these differences is severe ecological conditions, resulting in the accumulation of unusual metabolites as previously found in various Siberian plants [23,56,57,58].

The qualitative HPLC profiles of non-fermented and fermented leaves of *E. angustifolium* were similar; however, after quantification, we detected profound differences. The greatest changes were found for the basic ellagitannin oenothein B, the content of which after fermentation was reduced by 17.3 times (112.63 mg/g→6.53 mg/g); a reduction was also found for other ellagitannins but to a lesser degree. The gallic and ellagic acid content simultaneously increased by 6.4 and 3.9 times, respectively, in contrast to flavonoids and hydroxycinnamates, the content of which reduced slightly. This means that fermentation of *E. angustifolium* leaves affected mainly the ellagitannins and derivatives of gallic and ellagic acids; moreover, the breakdown of ellagitannins led to the release of free gallic and/or ellagic acids. Similar processes have been previously described for the hydrolytic cleavage [59,60] and microbial transformation of ellagitannins [40,61].

Some discrepancies in the level of HPLC-detected phenolic compounds before and after fermentation should be noted. The total content of detectable phenolics in non-fermented leaves of *E. angustifolium* was 242.27 mg/g but decreased to 103.70 mg/g after fermentation. The observed increase of gallic and ellagic acids was not so significant and, therefore, cannot explain the 57% loss of phenolics. That is why it is necessary to remember to discover a new post-fermentation metabolite, melanoidin, with a high content (145.31 mg/g) because the ellagitannins and melanoidin are most likely linked through biochemical reactions that originated in *E. angustifolium* leaves during fermentation. There are no data about the melanoidin of *E. Angustifolium;* therefore, we isolated and studied the physicochemical properties of the new pigment from fermented willowherb leaves.

### 3.2. Melanoidin from Fermented E. angustifolium Leaves: Physico-Chemical and Spectral Characteristics

#### 3.2.1. Isolation of *E. angustifolium* Melanoidin and General Characteristics

To isolate the dark pigment that causes the browning of *E. angustifolium* leaves, melanoidin, we used the sequential pre-extraction of plant raw materials with various solvents (hexane, ethyl acetate, and methanol), which made it possible to remove most of the low-molecular-weight compounds. Next, the remaining plant tissue was treated with hot water, which extracted the water-soluble melanoidins well [62], and the resulting extract after dialysis was precipitated with ethanol to remove polysaccharides and proteins. Melanoidin was precipitated from the mother liquor after acidification, which led to the production of a dark-coloured precipitate with a yield of 12.5% of dry fermented leaf weight.

**Figure 3:**
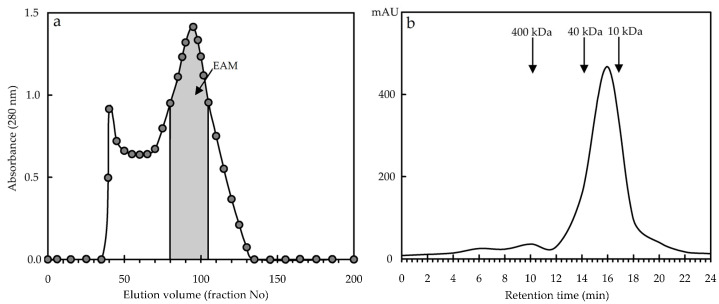
The elution profile of the total melanoidin fraction of *E. angustifolium* fermented leaves on a Sephadex G-150 column (**a**) and high-performance gel permeation chromatography with photodiode array detection (HPGPC-PDA) profile of purified melanoidin (EAM) (**b**).

After three-fold purification by reprecipitation, crude melanoidin was treated with 20% HCl, which destroys amine bonds without affecting the main melanoidin structure [63]. The use of gel permeation chromatography (GPC) on Sephacryl 300-HR showed that crude melanoidin is a heterogeneous drug containing a minor high-molecular-weight component and a dominant polymer in the molecular weight range of 10–20 kDa (Figure 3a). Multiple preparative GPC allowed the isolation of a target fraction of purified melanoidin (EAM) with a yield of 14% of crude melanoidin and a molecular weight (GP-HPLC) of about 1.4 × 10^4^ Da (Figure 3b).

The EAM sample did not contain carbohydrates or proteins but gave positive reactions with FeCl_3_, AgNO_3_, a discoloration reaction with H_2_O_2_ and KMnO_4_, and the dithionite-ferricyanide test, which is typical for melanoidins [64]. The solubility of EAM in organic solvents was nil but water and alkaline liquids (NaOH, KOH, LiOH, and NH_4_OH) were good dissolvents. Functional group analysis showed the presence of free carboxylic groups (4.29 ± 0.04%), phenolic hydroxyls (1.27 ± 0.02%), and pyrocatechol hydroxyls (5.63 ± 0.06%) in EAM, which (with a known molecular weight of EAM) means approximately 12 carboxylic groups, 10 phenolic hydroxyls, and 23 pyrocatechol hydroxyls per molecule.

#### 3.2.2. Elemental Composition

The elemental composition of EAM was 58.3% carbon, 4.3% hydrogen, 37.1% oxygen, and 0.2% nitrogen (Table 3). The known data on plant melanoidins indicate similar data for melanoidins formed after fermentation of the leaves or herb of plants such as *Camellia sinensis* [10] and *Orthosiphon stamineus* [12].

Melanoidins of non-fermentation origin typical for the original seed coats, fruit shells, or root epidermis from *Castanea mollissima* [65], *Echinacea purpurea* [66], *Nigella sativa* [67], *Randia echinocarpa* [68], *Sesamum indicum* [69], and *Helianthus anuus* [70] showed slightly different values. The value of the atomic ratio H/C (0.89) indicated the predominance of aromatic fragments in the EAM structure, and the value of O/C (0.48) showed the presence of oxygen functional groups.

**Figure 4:**
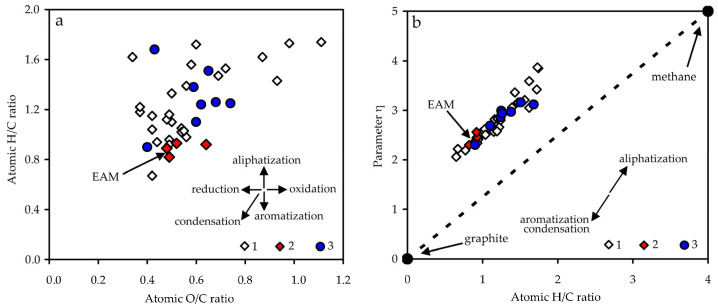
Van Krevelen diagram for known melanoidins (**a**) and atomic H/C as a function of parameter η (**b**). 1—fungal melanoidins; 2—plant melanoidins of fermentation origin; 3—plant melanoidins of non-fermentation origin.

A graphical representation of the dependence of O/C and H/C atomic ratios known as the Van Krevelen diagram allows us to compare the levels of aliphatization/aromatization (condensation) and reduction/oxidation of organic matter [71]. Using literature data to construct the Van Krevelen diagram, we found that plant melanoidins were similar to fungal melanoidins; however, there were certain differences (Figure 4a). Plant melanoidins of fermentation origin wre located in the zone of more aromatic and condensed compounds compared with fungal melanoidins, while melanoidins of non-fermentation origin were characterized as more aliphatic and oxidized substances, similar to fungal melanoidins.

The structural parameter η is a character independent of the molecular mass of a substance, which showed the lowest value for graphite (1; condensed/aromatic nature) and the highest for methane (5; saturated/aliphatic nature) [72]. The value of η for EAM was 2.36, demonstrating a medium level of condensation/aromatization. The known plant melanoidins of fermentation origin had a η value of 2.30–2.56, and melanoidins of non-fermentation origin had a value of 2.30–3.16 (Table 3). A previous study of fungal melanoidins showed a linear relationship between the atomic ratio H/C and the structural parameter η [62]. Plant melanoidins were not exceptional and were positioned in the same linear region of the H/C–η diagram (Figure 4b).

Another independent structural parameter, δ, is an unsaturation criterion with a value ranked in the range from −12.5 (methane) to 16.67 (graphite) [72]. The melanoidin of *E. angustifolium* had a δ value of 5.4, close to those of *Orthosiphon stamineus* melanoidin (5.6) [12] and black tea melanoidins (4.5–4.8) [10] with a high level of aromaticity. Most melanoidins of non-fermentation origin showed smaller δ values (1.3–4.0), which is typical for the more aliphatic phenolic polymers.

#### 3.2.3. UV-Vis Spectroscopy

**Figure 5:**
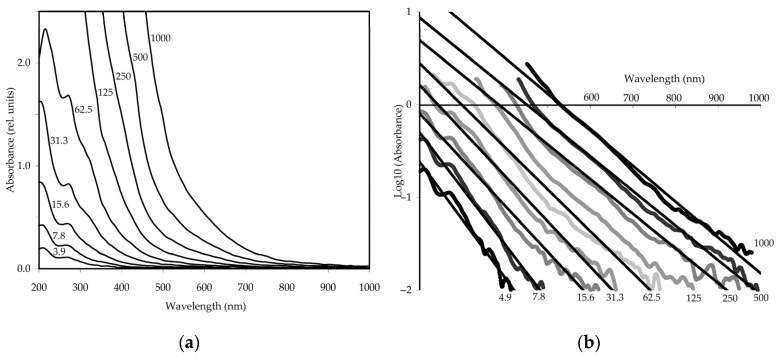
UV-Vis spectra of *E. angustifolium* melanoidin (EAM) in concentration range 3.9–1000 μg/mL (**a**) and diagrams of the wavelength–logarithm relationship of absorbance in the concentration range 3.9–1000 μg/mL (**b**).

The absorption spectrum of EAM contained two separate bands at 205 and 270 nm and a weak shoulder at 320 nm (Figure 5a). However, this apparent simplicity masks the extreme complexity of the spectrum. Based on the currently available data on the structure of melanoidins, their spectra in the first approximation should be the additive sum of the absorptions of many chromophore systems. The bands at 205 and 270 nm were B-band and C-band caused by A_1g_’→B_1u_’ and A_1g_’→B_2u_’ transitions, respectively [73], and the redshift of the spectrum compared to that of benzene was due to the influence of substituents, such as hydroxyls, alkyls, etc. [74].

The appearance of a shoulder in the region of 320 nm (K-band) was a result of elongation of the π-conjugation chain [75] while the asymmetric shape of these bands was due to the overlapping of various structural fragments. The presence of weak bands in the visible spectral region may be caused by the n→π* transitions of lone pairs of electrons from the oxygen of carbonyl groups.

The general shape of the absorption spectrum of neutral EAM solutions of various concentrations (3.9–1000 μg/mL) remained unchanged and the influence of alkaline solvents was weak. There was a slight decrease in the intensity of the B-band and a weak hypsochromic shift of it to 220 nm, while the C-band and K-band demonstrated an increase in intensity and bathochromic shifts to 272 and 330 nm, respectively. All these changes were due to the ionization of phenolic hydroxyls in an alkaline medium [76].

Some spectral characteristics of EAM were studied, such as the logarithm of the absorbance–wavelength linearity [12], chromatic coefficient E_465_/E_665_ [77], and the colour value (E^1%^_1 cm_) [64], which are usually used to characterize the chromatic properties and aromaticity level of melanoidins. The relationship of the logarithm of absorbance versus wavelength for EAM at pH 12.0 was linear (Figure 5b) as for most melanoidins [78]. The linearity of this relationship was stable independently of the melanoidin concentration; however, solutions with a higher concentration had better linearity in the long-wavelength region while less concentrated solutions showed better linearity at short wavelengths. The values of the determination coefficient (r^2^) were 0.9671–0.9904 for EAM solutions with a concentration range of 3.9–1000 µg/mL.

The logarithmic slope of absorbance for solutions with different concentrations was less than 0.0070 and varied from 0.0037 to 0.0062. The chromatic coefficient E_465_/E_665_ of EAM was low (6.71 ± 0.14), indicating a small number of aliphatic fragments and a high content of aromatic substituents in melanoidin [77]. The colour value of EAM (E^1%^_1 cm_) at 190 nm was 507 ± 10, higher than that of melanoidins from *Orthosiphon stamineus* (81) [12], *Osmanthus fragrans* (60) [64], and the general pigment (45) [79], which means a richer colour of EAM solutions.

#### 3.2.4. Fourier-Transform Infrared (FT-IR) Spectroscopy

**Figure 6:**
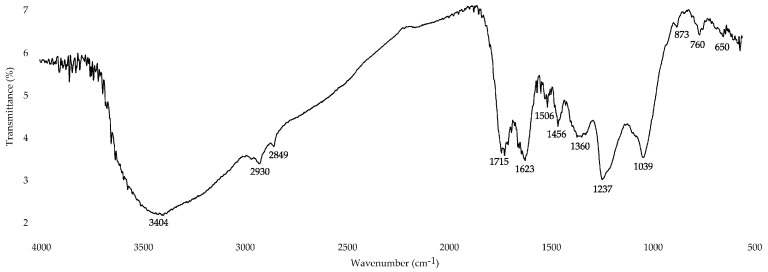
Fourier-transform infrared spectrum of *E. angustifolium* melanoidin (EAM).

The FT-IR spectrum of EAM (Figure 6) showed intense bands of hydroxyl groups at 3404 cm^−1^ as well as bands in the region 1000–1800 cm^−1^ caused by variously substituted C-H, C-O, and C-N functional groups [80]. In this spectral range, some specific bands were due to the presence of free carboxyl groups (1715 cm^−1^); aromatic fragments C=C and C=O (1623 cm^−1^); aromatic C-C groups and an amide II band (1506–1522 cm^−1^); aliphatic groups C-H and phenolic hydroxyls (1456 cm^−1^); variously substituted fragments C-N, N-H, and C-H (1360 cm^−1^); phenolic carbonyls and C-H (1237 cm^−1^); and ester bonds C-O-C and phenolic groups C-O (1039 cm^−1^) [12,42].

As for the vibrations in the range of 1506–1522 cm^−1^, they resulted from the system of aromatic rings and COOH groups not from amide II, due to the low nitrogen content in the EAM. Signals in the region below 880 cm^−1^ were caused by the aromatic protons of the melanoidin skeleton, and the weak bands at 2849–2970 cm^−1^ were caused by the aliphatic fragments C=H, CH_2_, and CH_3_, which have a low content [80].

In general, the FT-IR spectroscopy data confirmed the information about the aromatic/condensed nature of EAM found after elemental composition analysis and UV-Vis spectroscopy.

#### 3.2.5. NMR Spectroscopy

**Figure 7:**
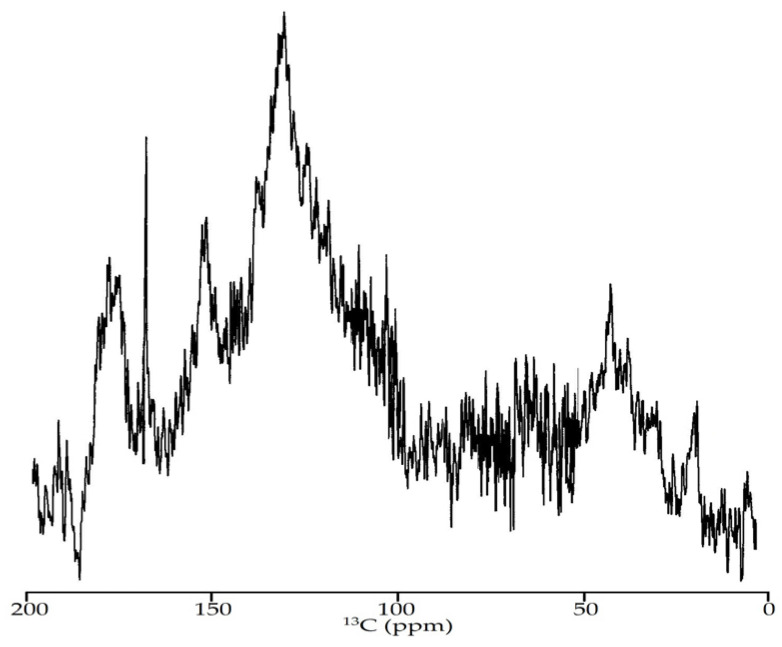
The NMR ^13^C spectrum of *E. angustifolium* melanoidin (EAM).

Further study of EAM was realized using ^13^C NMR spectroscopy. There have been few studies on the ^13^C NMR spectra of melanoidins, and those have been carried out for a small number of polymers [81,82]. In our case, the NMR spectrum of EAM contained a set of difficult to identify signals, which is typical for compounds with an irregular structure (Figure 7).

The general methodology for analysing the ^13^C NMR spectra of melanoidins is to determine the type of spectrum and the integral intensities of separate parts of it. For this, the total spectrum is conventionally divided into seven sections responsible for the presence of specific functional groups in the macromolecule, such as carboxyls or carbonyls (220–160 ppm), aromatic CO-R and/or CN-R groups (160–140 ppm), aromatic C-H; groups and C-2/C-6 atoms of phenolic acid fragments (140–110 ppm), anomeric hexose carbons and C-2/C-6 atoms of phenolic acid fragments (110–90 ppm), hexose C-2–C-5 atoms and C_α_ atoms of amino acids (90–60 ppm), methoxyl and hexose C-6 atoms (60–45 ppm), and methylenic groups of aliphatic fragments and methyls (45–0 ppm) [83].

The ^13^C NMR spectrum of EAM showed a highly intense aromatic fragment region (160–110 ppm, 52%) and low intensity for aliphatic structures (60–0 ppm, 24%) (Table 4). The spectral region 220–160 ppm demonstrated intensive peaks (16%), thus, confirming the presence of carboxyl groups.

Previously, the closest results were obtained for melanoidin from the micromycete *Inonotus obliquus* [62] with a high content of aromatic fragments (57%) but a high concentration of carboxyl groups (27%), as well as for *Castanea mollissima* melanoidins [65] with an aromatic fragment content of 20–56%. There is also information known about the melanoidins from some micromycetes, such as *Hendersonula toruloidea*, *Trichoderma harzianum* and *Ulocladium atrium,* with a low level of aromaticity (17–33%) [83]. Despite the lack of scientific data on ^13^C NMR spectroscopy of natural melanoidins, it can be assumed that the spectral characteristics are specific for different species.

#### 3.2.6. Alkaline Destruction

To understand the nature of the non-condensed fragment of *E. angustifolium* melanoidin, we used alkaline destruction of EAM followed by the HPLC-MS identification of cleavage products. It is generally estimated that the condensed nucleus of melanoidins is stable in alkaline media and only surface aromatic fragments can degrade and give some information about melanoidin structure.

Ten substances were found as the products of the alkaline destruction of EAM, including basic 3,4-dihydroxybenzoic acid (protocatechuic acid), 3-methoxy-4-hydroxybenzoic acid (vanillic acid), 4-hydroxybenzoic acid, 4-methoxybenzoic acid (*p*-anisic acid), and 3-hydroxybenzoic acid in the ratio 82:7:6:3:1, and trace compounds, such as 3-methoxybenzoic acid (*m*-anisic acid), benzoic acid, 3,4-dimethoxybenzoic acid (veratric acid), 3,5-dihydroxybenzoic acid, and 2,6-dihydroxybenzoic acid (γ-resorcylic acid) (Table 5). The results obtained are in good agreement with the early data on functional group content obtained by the titrimetric and spectrophotometric methods.

**Figure 8:**
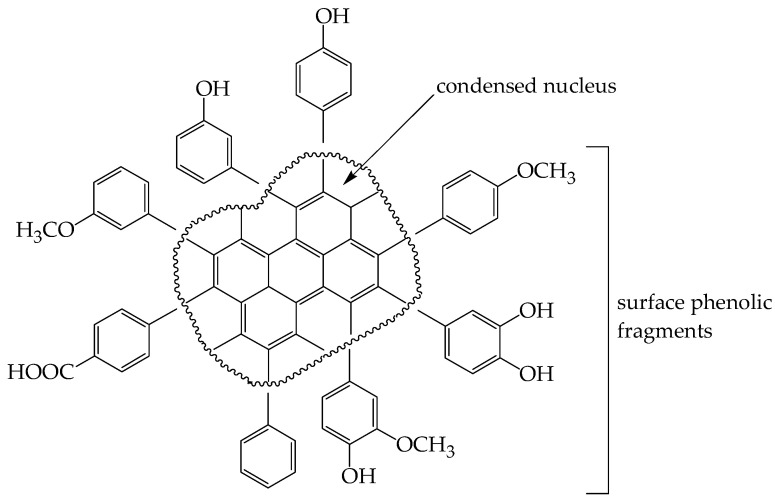
Hypothetical structure of *E. angustifolium* melanoidin EAM.

The studies performed indicate that EAM is a high-molecular-weight irregular phenolic polymer with a molecular weight of 1.4 × 10^4^ Da and a low nitrogen content. The main part of the EAM molecule is characterized by high aromaticity and contains sections of condensed benzene rings. The ‘surface’ of melanoidin is dotted with variously substituted hydroxy/methoxy benzoyl fragments with the domination of dihydroxyl and monohydroxyl residues, as well as free carboxyl groups (Figure 8).

The known data about plant melanoidins do not provide a clear sense of the structure of the melanoidin macromolecule [84,85]. In the absence of exact information, we can only assume that the structure proposed by us satisfies the physicochemical and spectral data obtained in our study.

### 3.3. Antioxidant Potential of E. angustifolium Melanoidin: In Vitro Activity and Gastrointestinal/Colonic Stability

The known protective role of melanoidins against free radicals, radiation, and toxic metals [86,87,88] requires us to investigate the antioxidant potential of EAM as a possible radical scavenger and metal chelator. It led us to use eight well-known antioxidant assays to study the scavenging properties against 2,2-diphenyl-1-picrylhydrazyl radicals (DPPH^•^), 2,2′-azino-bis(3-ethylbenzothiazoline-6-sulfonic acid) cation radicals (ABTS^•+^), *N*,*N*-dimethyl-*p*-phenylenediamine radicals (DMPD^•+^), superoxide anion radicals, hydroxyl radicals, and chloride radicals, as well as the nitric oxide scavenging potential and ferrous (II) ion chelating activity [89]. The bioactivity of oenothein B, the dominant antioxidant of the non-fermented herb of *E. angustifolium* [22], was also assessed against the activity of Trolox as a standard reference antioxidant [89].

Three synthetic free radicals, DPPH^•^, ABTS^•+^, and DMPD^•+^, were unstable when reacting with EAM (Table 6). The half-maximal scavenging concentrations (IC_50_) of EAM were 5.29–14.86 μg/mL, very close to those of Trolox (3.46–50.89 μg/mL) and oenothein B (8.61–25.33 μg/mL). Significant scavenging activity against inorganic radicals (superoxide anion radicals, hydroxyl radicals, and chloride radicals) was found for EAM and oenothein B, with a more pronounced potential of EAM. Both *E. angustifolium* components demonstrated effective inactivation of nitric oxide (II) molecules and chelation of ferrous (II) ions, outperforming the Trolox by many times. Finally, we concluded that EAM is an effective scavenger of free radicals and a chelator of metal ions comparable with the known antioxidant ellagitannin, oenothein B [22].

A basic possible reason for the high radical scavenging and metal chelating efficiency of EAM is the specific polymeric melanic structure in which the phenolic net can act as a one-dimensional semiconductor and electron acceptor [88]. The movable balance between the catechol, semiquinone, and quinine forms of melanoidin provides high-speed redox processes and a neutralizing effect on the free radicals and reactive oxygen forms [90]. Although this has not been discussed previously, the surface functional groups of melanoidins could act as metal chelators and electron conductors.

The high content of *o*-dihydroxy-phenolic functions on the EAM surface can also be considered as an increasing factor for antioxidant activity [30]. These electronic properties lead to the strong inhibitory effect of various melanoidins on lipid peroxidation [91,92] and the protection of living cells against oxidative damage [93]. Known plant melanoidins with antioxidant activity have been isolated from black tea [10], Java tea [12], sunflower [70], and some other species. The melanoidin from fermented willowherb is another example of a useful polymeric bioactive pigment.

In that regard, we can explain the highly similar TAC values for non-fermented and fermented samples of *E. angustifolium* and the similar antioxidant activity of both oenothein B and EAM. The fermentation of *E. angustifolium* resulted in a reduction in the content of the antioxidant ellagitannin and simultaneous accumulation of the pigment melanoidin with strong antioxidant properties. Consequently, one active compound is replaced by another, which ultimately compensates for the total activity of the sample.

**Figure 9:**
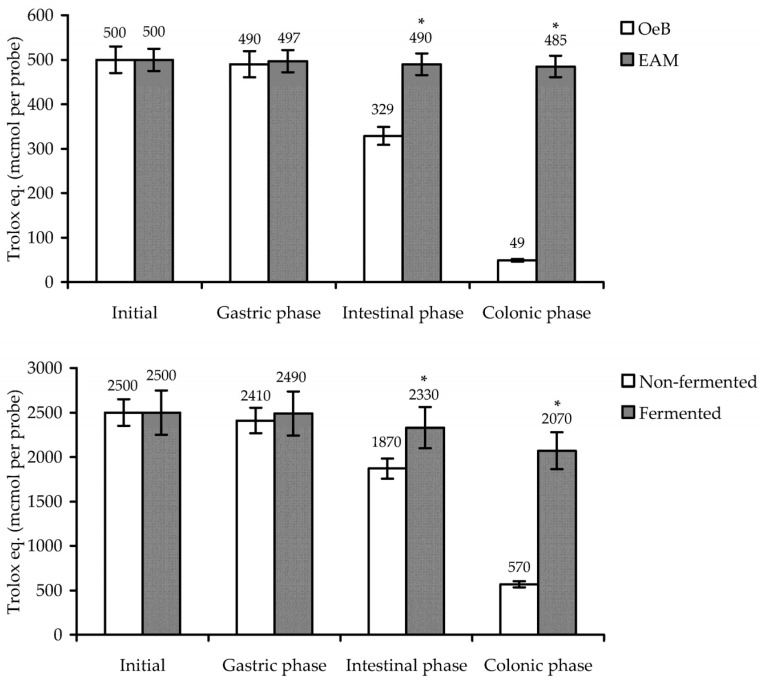
The trolox-equivalent content in oenothein B and *E. angustifolium* melanoidin (EAM) solutions, and *E. angustifolium* decoctions (non-fermented and fermented samples) after in vitro treatment by the simulated gastric medium (gastric phase), intestinal medium (intestinal phase) and after 72 h incubation with gut microbiota (colonic phase). * *p* < 0.05 vs. non-fermented group.

The high bioactivity of natural compounds is not always an indicator of effectiveness in a living organism, since digestion processes can harm the chemical stability of compounds [94]. Phenolic compounds in most cases are considered digestically stable but the separate organic groups, such as glycosides and ellagitannins, undergo significant changes, resulting in a drastic decrease of bioactivity [95]. We studied the gastrointestinal stability of EAM antioxidant activity using an in vitro assay including simulated phases of gastric, intestinal, and colonic digestion (Figure 9).

The final loss of Trolox-equivalent content in the EAM probe after the colonic phase of digestion was 3% of the original content, demonstrating good stability of EAM in simulated gastrointestinal fluids. By comparison, the oenothein B activity fluctuated considerably beginning in the intestinal phase of digestion, showing a 34% loss, followed by a dramatic fall in the colonic phase, with 90% loss. This phenomenon is explained by the poor stability of ellagitannins in the alkaline media of intestinal fluids [59] and the microbial degradation of ellagitannins to urolithins [61].

The revealed features of EAM and oenothein B stability directly affect the activity of extracts containing melanoidin and ellagitannin because of their dominant content. We examined the variation of the Trolox-equivalent content in decoctions of non-fermented and fermented samples of *E. angustifolium* during gastrointestinal digestion (Figure 9). In both cases, a reduction of activity was observed; however, the non-fermented sample showed 77% loss in contrast to the fermented sample with 17% loss of activity. The results obtained indicate that the fermented leaves of *E. angustifolium* have better antioxidant properties during digestion.

Despite the lack of information about the variation in antioxidant activity of fermented *E. angustifolium* in simulated gastrointestinal/colonic experimental models, we know that aerobic and anaerobic fermentation does not lead to significant changes in the activity of willowherb extracts [13]. The content of Trolox equivalents in fermented *E. angustifolium* leaves was in the range 462–488 mmol/100 g of dry weight vs. 386 mmol/100 g in non-fermented plants. Similar results have been obtained after the application of solid-phase fermentation [14,15]. Previously, this phenomenon was explained by a slight increase in low-molecular-weight antioxidants, but after our research, we can say that the reason lies in the accumulation of melanoidin.

The high level of melanoidin in the plant (145.31 mg/g) and its excellent antioxidant properties suggest that the polymeric pigment is the basic antioxidant in the fermented leaves of *E. angustifolium*. Still, we should consider the possibility of the additional impact of other active phenolics as flavonoids, and gallic and ellagic acids; however, their content in *E. angustifolium* leaves was much lower as was their importance. The known plant polymeric melanoidins provide the antioxidant properties of various foods like black tea [10], coffee [96], sesame [97], and oats [98], considered effective functional products recommended for everyday use. Fermented leaves of *E. angustifolium* are a good source of the antioxidant melanoidin and, thus, requires more in-depth study as a new functional product.

## 4. Conclusions

In the present study, the first comparative investigation of low- and high-molecular-weight phenolics was realized in non-fermented and fermented leaves of *E. angustifolium* and the new plant pigment of melanoidin nature was isolated and characterized. The results indicated that EAM is a polymer with an irregular and complex structure similar to that of known pigments from other fermented foods, such as black tea and Java tea. The specific structure of the polymer provides its high biological activity as an antioxidant and metal chelator and gastrointestinal and colonic stability in simulated conditions. The above demonstrates that the new melanoidin provides the high antioxidant activity of the fermented leaves of *E. Angustifolium,* as with other phenolics found in the plant.

Our findings have practical benefits in difficult situations, such as the COVID-19 pandemic, due to the new data that oxidative stress is a crucial factor aggravating COVID-19 severity [99]. The levels of basic internal antioxidants of non-enzymatic and enzymatic nature in COVID-19 patients are significantly lower, negatively influencing the prognosis of disease cure [100]. The documented cases of drug-induced oxidative stress after application of chloroquine and/or hydroxychloroquine make it more difficult to treat COVID-19 patients [101]. New recommendations for COVID-19 therapy relate to the intake of drugs, nutraceuticals, and food supplements that increase the antioxidant defence of the human organism [99,102]. In light of these observations, the high antioxidant potential of the fermented leaves of *E. angustifolium* and new melanoidin make it useful for the treatment and prevention of oxidative stress during the various diseases.

## Figures and Tables

**Table 1:** The chemical composition of *Epilobium angustifolium* before and after fermentation ± S.D.

Compound Group	*E. angustifolium* before Fermentation	*E. angustifolium* after Fermentation
Extractives (solvent water), % ^a^	28.42 ± 0.84	38.53 ± 1.15
Extractives (solvent methanol), % ^a^	41.20 ± 1.38	49.63 ± 1.47
Lipids, mg/g ^b^	43.70 ± 1.29	41.61 ± 1.21
Carotenoids, mg/g ^b^	0.41 ± 0.01	0.39 ± 0.01
Chlorophylls, mg/g ^b^	8.65 ± 0.17	trace
Proteins, mg/g ^b^	22.14 ± 0.45	14.63 ± 0.28
Free amino acids, mg/g ^b^	20.10 ± 0.40	37.82 ± 0.74
Water-soluble polysaccharides, mg/g ^b^	28.93 ± 0.84	19.11 ± 0.57
Mono-/disaccharides, mg/g ^b^	35.18 ± 1.05	56.63 ± 1.12
Titratable acidity, mg/g ^b^	18.59 ± 0.37	25.61 ± 0.51
Total phenolics, mg/g ^b^	277.15 ± 6.73	252.11 ± 6.04
Flavonoids, mg/g ^b^	52.73 ± 1.21	50.61 ± 1.19
Tannins, mg/g ^b^	183.11 ± 3.66	12.52 ± 0.32
Melanoidin, mg/g ^b^	not detected	145.31 ± 4.35

^a^ g per 100 g of Dry Plant Weight (DPW); ^b^ mg per g DPW.

**Table 2:** Compounds **1**–**59** were found in *E. angustifolium* leaves before and after fermentation.

No	t_R_, min	Compound ^a^	Previous Data ^b^	[M − H]^−^, *m*/*z* ^c^	MS/MS, *m*/*z*	Content in Leaves, mg/g of Dry Plant Weight ± S.D.	
						before Fermentation	after Fermentation
**1**	2.27	1-*O*-Galloyl-^βD^Glc*p* ^S^ (glucogallin) [27]		331	169	0.83 ± 0.02	traces
**2**	2.53	Gallic acid *O*-Hex ^L^ [27]		331	169	0.96 ± 0.02	traces
**3**	3.28	Gallic acid ^S^ [42]	[43]	169		2.37 ± 0.04	15.27 ± 0.30
**4**	4.51	Gall-HHDP-Hex_2_ ^L^ [43,44,45]		795	633, 481, 463, 301	0.57 ± 0.01	traces
**5**	4.77	Gall-HHDP-Hex_2_ ^L^ [43,44,45]		795	633, 481, 463, 301	0.73 ± 0.02	traces
**6**	5.01	Gall-HHDP-Hex ^L^ [43,44,45]	[43]	633	481, 463, 301	1.67 ± 0.03	0.22 ± 0.00
**7**	5.32	Oenothein B ^S^ [43,46]	[43]	1567, 783 *	765, 301	112.62 ± 2.36	6.53 ± 0.12
**8**	5.57	Oenothein A ^L^ [43,46]	[45]	1175 *	765, 301	29.63 ± 0.56	2.77 ± 0.05
**9**	5.91	Tetrameric tellimagrandin I ^L^ [45,47]	[45]	1044 **	765, 301, 275	10.56 ± 0.20	0.93 ± 0.02
**10**	6.24	Pentameric tellimagrandin I ^L^ [45,47]	[45]	1035 **	765, 301, 275	6.73 ± 0.11	4.22 ± 0.09
**11**	6.49	Hexameric tellimagrandin I ^L^ [45,47]	[45]	1567 **	765, 301, 275	3.25 ± 0.06	1.20 ± 0.02
**12**	6.68	Heptameric tellimagrandin I ^L^ [45,47]	[45]	1828 **	765, 301, 275	1.04 ± 0.02	0.83 ± 0.02
**13**	7.37	Ellagic acid *O*-Hex_3_ ^L^ [48]		787	625, 463, 301	0.43 ± 0.01	traces
**14**	7.51	1-*O*-Ellagoyl-gentiobiose ^S^ (amritoside) [48]		625	463, 301	1.12 ± 0.02	0.37 ± 0.01
**15**	7.93	Di-ellagoyl *O*-Hex_3_ ^L^ [48]		1071	909, 787, 625, 463, 301	0.20 ± 0.00	traces
**16**	8.17	1,6′-Di-*O*-ellagoyl-gentiobiose ^S^ (granatoside A) [48]		909	463, 341, 301	0.43 ± 0.01	traces
**17**	8.53	1-*O*-Ellagoyl-^βD^Glc*p* ^S^ [48]		463	301	0.18 ± 0.00	traces
**18**	9.36	Ellagic acid ^S^ [43]	[43]	301		2.83 ± 0.05	10.91 ± 0.20
**19**	9.56	1,6′-Di-*O*-ellagoyl-^βD^Glc*p* ^S^ (granatoside B) [48]		747	463, 301	2.07 ± 0.05	0.31 ± 0.00
**20**	10.21	Ellagic acid *O*-Hex_3_-*O*-Ac ^L^ [28,44]		829	787, 625, 463, 301	traces	traces
**21**	10.47	Ellagic acid_2_ *O*-Hex_3_-*O*-Ac ^L^ [28,44]		1113	1071, 909, 787, 625, 463, 301	0.37 ± 0.01	traces
**22**	12.01	Ellagic acid *O*-methyl ester *O*-Hex ^L^ [28,44]		477	315, 301	traces	traces
**23**	12.52	Ellagic acid di-*O*-methyl ester *O*-Hex ^L^ [28,44]		491	329, 301	traces	traces
**24**	14.90	Ellagic acid_2_ *O*-methyl ester *O*-Hex ^L^ [28,44]		761	477, 315, 301	traces	traces
**25**	17.54	Ellagic acid_2_ di-*O*-methyl ester *O*-Hex ^L^ [28,44]		775	477, 315, 301	0.29 ± 0.00	0.14 ± 0.00
**26**	18.95	Ellagic acid tri-*O*-methyl ester *O*-Hex ^L^ [28,44]		505	343, 301	traces	traces
**27**	20.99	1-*O*-Sinapoyl-^βD^Glc*p* ^S^ [49]		385	223	traces	traces
**28**	21.14	6-*O*-Sinapoyl-^βD^Glc*p* ^S^ [49]		385	223	traces	traces
**29**	21.47	1-*O*-Caffeoyl-^βD^Glc*p* ^S^ [49]		341	179, 135	traces	traces
**30**	22.72	1-*O*-Caffeoylquinic acid ^S^ [50]		353	191, 179, 173, 135	traces	traces
**31**	25.27	4-*O*-Caffeoylquinic acid ^S^ [50]		353	191, 179, 173, 135	8.68 ± 0.16	8.21 ± 0.16
**32**	25.48	Caffeic acid_2_ *O*-Hex ^L^ [51]		503	341, 179, 135	traces	traces
**33**	25.53	Myricetin *O*-HexA-*O*-Hex-*O*-Gall_2_ ^L^ [52]		959	807, 655, 493, 317	traces	traces
**34**	25.72	Quercetin *O*-HexA-*O*-Hex-*O*-Gall_2_ ^L^ [52]		943	791, 639, 477, 301	traces	traces
**35**	25.93	Quercetin *O*-HexA-*O*-Hex_2_-*O*-Gall ^L^ [52]		953	801, 639, 477, 301	traces	traces
**36**	26.14	5-*O*-Caffeoylquinic acid ^S^ [50]	[43]	353	191, 179, 165	5.27 ± 0.11	4.63 ± 0.10
**37**	26.45	Myricetin *O*-HexA-*O*-Hex-*O*-Gall ^L^ [52]		807	655, 493, 317	traces	traces
**38**	26.58	Quercetin *O*-HexA-*O*-Hex-*O*-Gall ^L^ [52]		791	639, 477, 301	traces	traces
**39**	26.87	Myricetin *O*-Hex-*O*-Gall ^L^ [52]	[43]	631	479, 317	traces	traces
**40**	27.21	Quercetin *O*-Hex-*O*-Gall_2_ ^L^ [52]		767	615, 463, 301	traces	traces
**41**	27.80	Myricetin *O*-HexA ^L^ [45]	[45]	493	317	5.69 ± 0.11	5.43 ± 0.11
**42**	27.98	Myricetin-3-*O*-^βD^Gal*p* ^S^ [43]	[43]	479	317	0.36 ± 0.00	0.22 ± 0.00
**43**	28.49	Quercetin-3-*O*-(6″-*O*-Gall)-^β^^D^Gal*p* ^S^ [43]	[43]	615	463, 301	4.56 ± 0.09	4.02 ± 0.08
**44**	28.81	Myricetin-3-*O*-^αL^Rha*p* ^S^ (myricitrin) [43]	[43]	463	317	0.46 ± 0.01	0.31 ± 0.00
**45**	29.11	Quercetin-*O*-Rut ^S^ (rutin) [46]		609	463, 301	traces	traces
**46**	29.79	Quercetin-3-*O*-^β^^D^GlcA*p* ^S^ (miquelianin) [43]	[43]	477	301	31.65 ± 0.65	30.81 ± 0.62
**47**	30.02	Quercetin-3-*O*-^β^^D^Gal*p* ^S^ (hyperoside) [43]	[43]	463	301	0.93 ± 0.02	0.86 ± 0.02
**48**	30.54	Kaempferol *O*-Hex-*O*-Gall ^L^ [43]	[43]	599	447, 285	traces	traces
**49**	31.51	Quercetin-3-*O*-^α^^L^Ara*f* ^S^ (avicularin) [43]	[43]	433	301	1.98 ± 0.04	1.53 ± 0.03
**50**	32.63	Kaempferol-3-*O*-^β^^D^GlcA*p* ^S^ [43]	[43]	461	285	2.35 ± 0.05	2.21 ± 0.04
**51**	33.12	Quercetin-3-*O*-^α^^L^Rha*p* ^S^ (quercitrin) [43]	[43]	447	301	0.53 ± 0.01	0.50 ± 0.01
**52**	35.87	Kaempferol-3-*O*-^α^^L^Rha*p* ^S^ (afzelin) [43]	[43]	431	285	0.39 ± 0.00	0.26 ± 0.00
**53**	36.95	Ellagic acid 3-*O*-methyl ester ^S^ [28,46]		315	301	traces	traces
**54**	37.32	Ellagic acid 3,3′-di-*O*-methyl ether ^S^ [28,46]		329	301	traces	traces
**55**	38.73	Myricetin *O*-Hex-*O*-*p*Cou ^L^ [53]		625	479, 317	traces	traces
**56**	38.98	Quercetin-3-*O*-(6″-*O*-*p*Cou)-^β^^D^Glc*p* ^S^ (helichrysoside) [53]		609	463, 301	1.14 ± 0.02	1.01 ± 0.02
**57**	39.08	Kaempferol-3-*O*-(6″-*O*-*p*Cou)-^β^^D^Glc*p* ^S^ (tiliroside) [53]	[54]	593	447, 285	traces	traces
**58**	39.22	Quercetin *O*-Hex-*O*-*p*Cou-*O*-Ac [53]		651	609, 463, 301	traces	Traces
**59**	39.48	Kaempferol O-Hex-O-pCou-O-Ac [53]		635	593, 447, 285	traces	traces
Total content:							
gallic acid/gallic acid glycosides						4.16	15.27
ellagitannins						166.80	16.70
ellagic acid/ellagic acid glycosides						7.92	11.73
hydroxycinnamates						14.13	12.84
myricetin glycosides						6.51	5.96
quercetin glycosides						40.79	38.73
kaempferol glycosides						2.79	2.47
flavonol glycosides						50.09	47.16
phenolics						243.10	103.70

^a^ Compound identification was based on comparison of retention time, UV and MS spectral data with reference standard (^S^) or interpretation of UV and MS spectral data and comparison with literature data (^L^). ^b^ Reference for known data of compound presence in *E. angustifolium*. ^c^ * [M − 2H]^2−^. ** [M − 3H]^3−^. Abbreviation used: Ac—acetate; ^αL^Ara*f*—α-l-arabinofuranose; *p*Cou—*p*-coumaroyl; ^βD^Gal*p*—β-d-galactopyranose; ^βD^Glc*p*—β-d-glucopyranose; ^βD^GlcA*p*—β-d-glucuronopyranose; Gall—galloyl; Hex—hexose; HexA—hexuronic acid; ^αL^Rha*p*—α-l-rhamnopyranose.

**Table 3:** The elemental composition of *E. angustifolium* melanoidin (EAM) and selected known plant melanoidins.

Plant Source [Ref.]	Element Content, %				Atomic Ratio		Parameter η	Structural Parameter δ
	Carbon	Hydrogen	Oxygen	Nitrogen	Hydrogen/Carbon	Oxygen/Carbon		
*E. angustifolium* (EAM)	58.3	4.3	37.1	0.2	0.89	0.48	2.36	5.4
*Camellia sinensis* [10]	54.2	4.2	38.3	3.2	0.93	0.52	2.46	4.8
	49.8	3.8	42.5	3.8	0.92	0.64	2.56	4.5
*Orthosiphon stamineus* [12]	57.1	3.9	36.9	2.0	0.82	0.49	2.30	5.6
*Castanea mollissima* [65]	47.0	4.9	46.5	1.5	1.25	0.74	2.99	2.9
	52.5	4.8	41.8	0.9	1.10	0.60	2.69	4.0
	51.4	5.3	42.5	0.8	1.24	0.62	2.86	3.3
*Echinacea purpurea* [66]	50.0	7.0	29.1	13.0	1.68	0.43	3.12	1.3
*Nigella sativa* [67]	50.3	5.8	39.4	4.4	1.38	0.59	2.97	2.6
*Randia echinocarpa* [68]	48.3	6.1	41.8	3.7	1.51	0.65	3.16	2.0
*Sesamum indicum* [69]	47.6	5.0	43.4	3.7	1.26	0.68	2.94	3.1
*Helianthus anuus* [70]	61.1	4.6	32.5	1.7	0.90	0.40	2.30	5.6

**Table 4:** The relative intensity of ^13^C NMR signal in the various ranges of chemical shift of melanoidins (%).

Melanoidin Source [Ref.]	NMR ^13^C Spectrum Region, ppm						
	220–160	160–140	140–110	110–90	90–60	60–45	45–0
*E. angustifolium* (EAM)	16	17	35	8	5	5	14
*Castanea mollissima* [65]	10–13	6–21	14–35	39–68			8–13
*Inonotus obliquus* [62]	27	14	43	3	1	1	11
*Hendersonula toruloidea* [83]	15	8	16	9	18	11	20
*Trichoderma harzianum* [83]	15	3	9	5	17	12	39
*Ulocladium atrium* [83]	7	2	5	11	43	7	25

**Table 5:** The retention times (t), UV, mass spectral data, and relative content of cleavage compounds after the alkaline destruction of *E. angustifolium* melanoidin.

Compound	t, min	UV Spectrum, λ_max_, nm	ESI-MS Data, *m*/*z*	Relative Content, %
3,5-Dihydroxybenzoic acid	4.92	203, 249, 307	153	traces
3,4-Dihydroxybenzoic acid	5.22	203, 259, 293	153	82
2,6-Dihydroxybenzoic acid	6.34	203, 307	153	traces
4-Hydroxybenzoic acid	6.70	201, 255	137	6
3-Methoxy-4-hydroxybenzoic acid	7.57	202, 260, 288	167, 153	7
3-Hydroxybenzoic acid	7.93	202, 297	137	1
4-Methoxybenzoic acid	10.22	205, 268	151, 137	3
3,4-Dimethoxybenzoic acid	10.56	204, 258, 289	181, 167, 153	traces
Benzoic acid	11.42	225, 271	121	traces
3-Methoxybenzoic acid	12.81	200, 291	151, 137	traces

**Table 6:** The antioxidant activity of *E. angustifolium* melanoidin (EAM), oenothein B, and Trolox in eight in vitro assays.

Assay ^a^	EAM	Oenothein B	Trolox
DPPH^• b^	6.14 ± 0.14 ^i^	10.53 ± 0.21 ^iii^	8.50 ± 0.15 ^ii^
ABTS^•+ b^	5.29 ± 0.11 ^v^	8.61 ± 0.15 ^vi^	3.46 ± 0.07 ^iv^
DMPD^•+ b^	14.86 ± 0.30 ^vii^	25.33 ± 0.52 ^viii^	50.89 ± 0.99 ^ix^
O_2_^•− b^	12.69 ± 0.24 ^x^	37.18 ± 0.75 ^xi^	89.12 ± 1.62 ^xii^
^•^OH ^b^	15.26 ± 0.28 ^xiv^	28.63 ± 0.53 ^xv^	10.37 ± 0.18 ^xiii^
Cl^• c^	1293.73 ± 19.39 ^xvii^	1108.67 ± 22.10 ^xvii^	1000 ^xvi^
NO ^d^	27.20 ± 0.52 ^xviii^	35.11 ± 0.71 ^xix^	893.14 ± 26.79 ^xx^
FeCA ^e^	396.15 ± 9.91 ^xxiii^	293.15 ± 7.11 ^xxii^	40.27 ± 0.85 ^xxi^

^a^ DPPH^•^—scavenging of 2,2-diphenyl-1-picrylhydrazyl radicals; ABTS^•+^—scavenging of 2,2′-azino-bis(3-ethylbenzothiazoline-6-sulfonic acid) cation radicals; DMPD^•+^—scavenging of *N*,*N*-dimethyl-*p*-phenylenediamine radicals; O_2_^•−^—scavenging of superoxide anion radicals; ^•^OH—scavenging of hydroxyl radicals; Cl^•^—chloride radicals; and NO—scavenging of nitric oxide (II); FeCA—ferrous (II) ion chelating activity. ^b^ IC_50_, μg/mL. ^c^ mg Trolox eq./g. ^d^ IC_50_, mg/mL. ^e^ mg Fe^2+^/g. Averages ± standard deviation (S.D.) were obtained from five different experiments. Values with different numbers (i–xxiii) indicate statistically significant differences among groups at *p* < 0.05 by one-way ANOVA.

## Data Availability

Data is contained within the article or supplementary materials.

## Supplementary Materials

The following are available online at https://www.mdpi.com/article/10.3390/antiox10081300/s1, Table S1: Reference standards used for the qualitative and quantitative analysis by HPLC-MS, Table S2: Regression equations, correlation coefficients, standard deviation, limits of detection, limits of quantification and linear ranges for 31 reference standards.
