# Supplementary material for: Water-Soluble Melanoidin Pigment as a New Antioxidant Component of Fermented Willowherb Leaves (Epilobium angustifolium)
Source: Antioxidants (Basel). 2021 Aug 18;10(8):1300. doi: 10.3390/antiox10081300 (PMC8389334; doi:10.3390/antiox10081300)
Supplement: Supplementary file 1 [file antioxidants-10-01300-s001.zip › antioxidants-1335279-supplementary.pdf]

antioxidants

Supplementary Material

Water-soluble Melanoidin Pigment as a New Antioxidant Component of Fermented Willowherb Leaves (*Epilobium angustifolium*)

Daniil N. Olennikov [1,*], Christina S. Kirillina [2] and Nadezhda K. Chirikova [2]

[1] Laboratory of Medical and Biological Research, Institute of General and Experimental Biology, Siberian Division, Russian Academy of Science, 670047 Ulan-Ude, Russia
[2] Department of Biology, Institute of Natural Sciences, North-Eastern Federal University, 677027 Yakutsk, Russia; kristinakirillina22@gmail.com (C.S.K.), hofnung@mail.ru (N.K.C.)
* Correspondence: olennikovdn@mail.ru; Tel.: +8-902-160-06-27

Citation: Olennikov, D.N.; Kirillina, C.S.; Chirikova, N.K. Water-soluble Melanoidin Pigment as a New Antioxidant Component of Fermented Willowherb Leaves (*Epilobium angustifolium*). *Antioxidants* **2021**, *10*, 1300. https://doi.org/10.3390/antiox10081300

Academic Editor: María Pilar Almajano Pablos

Received: 26 July 2021
Accepted: 15 August 2021
Published: 18 August 2021

Publisher's Note: MDPI stays neutral with regard to jurisdictional claims in published maps and institutional affiliations.

Copyright: © 2021 by the authors. Licensee MDPI, Basel, Switzerland. This article is an open access article distributed under the terms and conditions of the Creative Commons Attribution (CC BY) license (http://creativecommons.org/licenses/by/4.0/).

Content

Table S1. Reference standards used for the qualitative and quantitative analysis by HPLC-MS.

Table S2. Regression equations, correlation coefficients (r^2), standard deviation (S_{YX}), limits of detection (LOD), limits of quantification (LOQ) and linear ranges for 31 reference standards.

Table S1. Reference standards used for the qualitative and quantitative analysis by HPLC-MS.

**Compound, formula**	**Purity (≥), %**	**Manufacturer (cat. No) or isolation reference** [a]	**Used for analysis of compounds (No in Table 2 or name in Table 5)**
1-*O*-Galloyl-β-D-glucopyranose 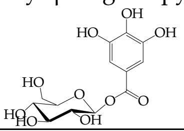	90	Sigma (69288)	QL: **1**, **2**. QT: **1**, **2**
Gallic acid 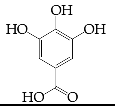	97	Sigma (G7384)	QL: **3**. QT: **3**
Oenothein B 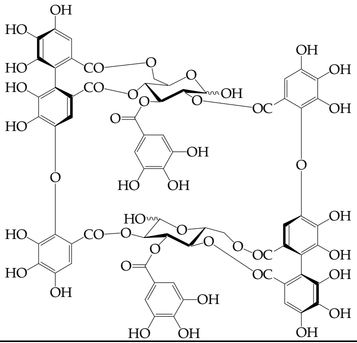	98	Biopurity (BP1809)	QL: **7**. QT: **7–12**
Amritoside = 1-*O*-ellagoyl-gentiobiose 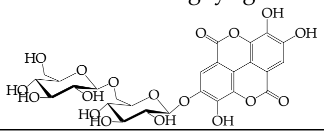	No data	Lab collection/isolated from *Punica granatum* [48]	QL: **14**
Granatoside A = 1,6′-di-*O*-ellagoyl-gentiobiose 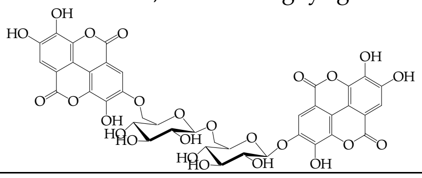	No data	Lab collection/isolated from *Punica granatum* [48]	QL: **16**
1-*O*-Ellagoyl-β-D-glucopyranoside 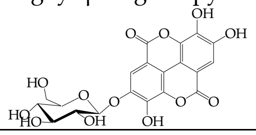	No data	Lab collection/isolated from *Punica granatum* [48]	QL: **17**
Ellagic acid 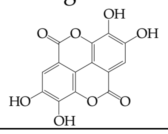	95	Sigma (E2250)	QL: **18**. QT: **4–6**, **13–19**, **20–26**
Granatoside B = 1,6-Di-*O*-ellagoyl-β-D-glucopyranoside 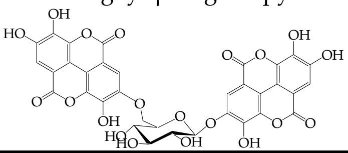	No data	Lab collection/isolated from *Punica granatum* [48]	QL: **19**

Table S1. *Cont*.

Compound, formula	Purity (>), %	Manufacturer (cat. No) or isolation reference [a]	Used for analysis of compounds (No in Table 2 or name in Table 5)
1-*O*-Sinapoyl-β-D-glucopyranoside 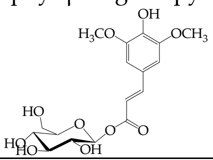	No data	Lab collection/isolated from *Panax ginseng* [49]	QL: **27**
6-*O*-Sinapoyl-β-D-glucopyranoside 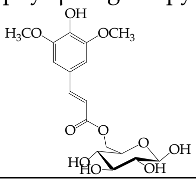	No data	Lab collection/isolated from *Panax ginseng* [49]	QL: **28**
1-*O*-Caffeoyl-β-D-glucopyranoside 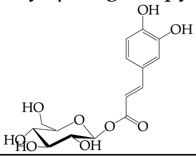	No data	Lab collection/isolated from *Panax ginseng* [49]	QL: **29**
1-*O*-Caffeoylquinic acid 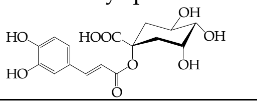	98	ChemFaces (CFN99121)	QL: **30**. QT: **30**
4-*O*-Caffeoylquinic acid 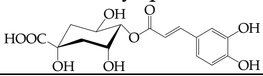	98	Sigma (65969)	QL: **31**. QT: **31**
5-*O*-Caffeoylquinic acid 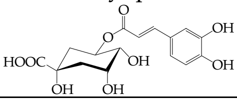	95	Sigma (C3878)	QL: **36**. QT: **36**
Myricetin-3-*O*-β-D-galactopyranoside 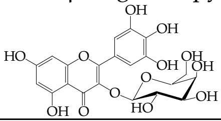	85	Sigma (SMB00249)	QL: **42**. QT: **33**, **37**, **39**, **41**, **42**, **55**
Quercetin-3-*O*-(6′′-galloyl)-β-D-galactopyranoside 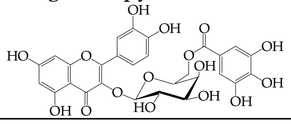	98	MCE (HY-N7024)	QL: **43**. QT: **34**, **35**, **38**, **40**, **43**
Myricitrin = myricetin-3-*O*-α-L-rhamnopyranoside 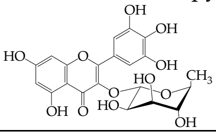	99	Sigma (91255)	QL: **44**. QT: **44**
Rutin = quercetin-3-*O*-rutinoside 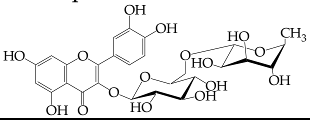	95	Sigma (R2303)	QL: **45**. QT: **45**

Table S1. *Cont.*

Compound, formula	Purity (>), %	Manufacturer (cat. No) or isolation reference [a]	Used for analysis of compounds (No in Table 2 or name in Table 5)
Miquelianin = quercetin-3-*O*-β-D-glucuronopyranoside 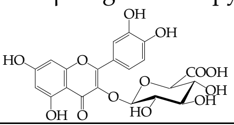	95	Sigma (00310590)	QL: **46**. QT: **46**
Hyperoside = quercetin-3-*O*-β-D-galactopyranoside 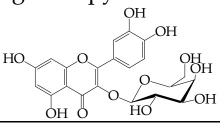	98	Extrasynthese (1027S)	QL: **47**. QT: **47, 56**, **58**
Avicularin = quercetin-3-*O*-α-L-arabinofuranoside 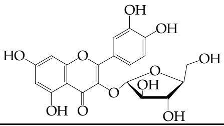	95	Sigma (75759)	QL: **49**. QT: **49**
Kaempferol-3-*O*-β-D-glucuronopyranoside 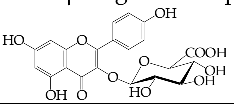	97	Sigma (79273)	QL: **50**. QT: **48**, **50**
Quercitrin = quercetin-3-*O*-α-L-rhamnopyranoside 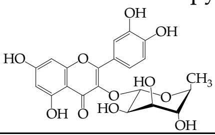	98	Sigma (740580)	QL: **51**. QT: **51**
Afzelin = kaempferol-3-*O*-α-L-rhamnopyranoside 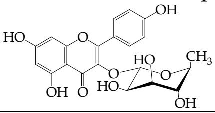	98	Sigma (PHL83864)	QL: **52**. QT: **52**
3-*O*-Methyl ellagic acid 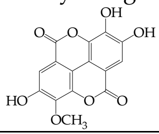	90	Sigma (PHL82590)	QL: **53**. QT: **53**
Ellagic acid 3,3′-dimethyl ether 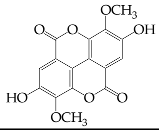	98	ChemFaces (CFN98217)	QL: **54**. QT: **54**
Helichrysoside = quercetin-3-*O*-(6′′-*O*-*p*-coumaroyl)-β-D-glucopyranoside 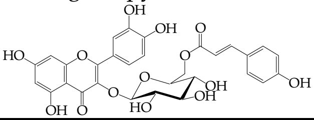	No data	Lab collection/isolated from *Spiraea salicicfolia* [53]	QL: **56**

Table S1. *Cont.*

Compound, formula	Purity (≥), %	Manufacturer (cat. No) or isolation reference [a]	Used for analysis of compounds (No in Table 2 or name in Table 5)
Tiliroside = kaempferol-3-*O*-(6′′-*O*-*p*-coumaroyl)-β-D-glucopyranoside 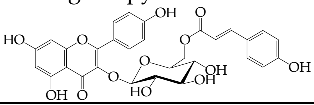	98	Sigma (79257)	QL: **57**. QT: **57**, **59**
Sinapic acid 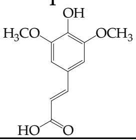	98	Sigma (D7927)	QT: **27**, **28**
Caffeic acid 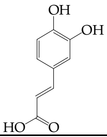	98	Sigma (C0625)	QT: **29**, **32**
3,5-Dihydroxybenzoic acid 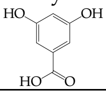	97	Sigma (D110000)	QL: 3,5-dihydroxybenzoic acid
3,4-Dihydroxybenzoic acid 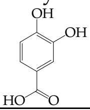	97	Sigma (37580)	QL: 3,4-dihydroxybenzoic acid
2,6-Dihydroxybenzoic acid 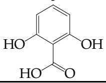	98	Sigma (D109606)	QL: 2,6-dihydroxybenzoic acid
4-Hydroxybenzoic acid 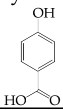	99	Sigma (240141)	QL: 4-hydroxybenzoic acid
3-Methoxy-4-hydroxybenzoic acid 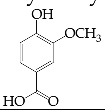	97	Sigma (H36001)	QL: 3-methoxy-4-hydroxybenzoic acid
3-Hydroxybenzoic acid 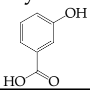	99	Sigma (H20008)	QL: 3-hydroxybenzoic acid
4-Methoxybenzoic acid 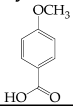	99	Sigma (117390)	QL: 4-methoxybenzoic acid
3,4-Dimethoxybenzoic acid 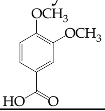	99	Sigma (D131806)	QL: 3,4-dimethoxybenzoic acid
Benzoic acid 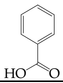	99.5	Sigma (242381)	QL: benzoic acid

Table S1. *Cont.*

Compound, formula	Purity (≥), %	Manufacturer (cat. No) or isolation reference [a]	Used for analysis of compounds (No in Table 2 or name in Table 5)
3-Methoxybenzoic acid 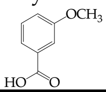	99	Sigma (117714)	QL: 3-methoxybenzoic acid

[a] Manufacturers list: Biopurity—Biopurity Phytochemicals Ltd (Chengdu, PRC); ChemFaces—ChemFaces (Wuhan, Hubei, PRC); Extrasynthese—Extrasynthese (Lyon, France); MCE—MCE Med Chem Express (Monmouth, NJ, USA); Sigma—Sigma-Aldrich (St. Louis, MO, USA). [b] QL—qualitative analysis; QT—quatitative analysis.

Table S2. Regression equations, correlation coefficients (r^2), standard deviation (S_{YX}), limits of detection (LOD), limits of quantification (LOQ) and linear ranges for 31 reference standards.

Compound	Ionization [a]	CE [b] (eV)	Regression equation [c]		r^2	S_{YX}	LOD/ LOQ (µg/mL)	Linear range (µg/mL)
			a	$b \cdot 10^6$				
1-*O*-Caffeoylquinic acid	N	-15	2.5394	-1.2360	0.9994	$0.45 \cdot 10^{-2}$	0.006/0.02	0.02–300.0
1-*O*-Galloyl-β-D-glucopyranose	N	-20	1.3586	-0.0663	0.9987	$9.69 \cdot 10^{-2}$	0.24/0.71	0.8–100.0
3-*O*-Caffeoylquinic acid	N	-15	2.4176	-1.5647	0.9994	$0.40 \cdot 10^{-2}$	0.005/0.02	0.02–300.0
3-*O*-Methyl ellagic acid	N	-35	0.9361	-0.4518	0.9870	$9.35 \cdot 10^{-2}$	0.32/1.00	1.0–100.0
4-*O*-Caffeoylquinic acid	N	-15	2.7365	-1.0690	0.9996	$0.51 \cdot 10^{-2}$	0.006/0.02	0.02–300.0
5-*O*-Caffeoylquinic acid	N	-15	2.9021	-1.4184	0.9998	$0.39 \cdot 10^{-2}$	0.004/0.01	0.02–300.0
Afzelin = kaempferol-3-*O*-α-L-rhamnopyranoside	N	-20	2.0859	-0.9171	0.9980	$6.18 \cdot 10^{-2}$	0.03/0.09	0.10–100.0
Avicularin = quercetin-3-*O*-α-L-arabinofuranoside	N	-20	1.4412	-0.6211	0.9930	$11.25 \cdot 10^{-2}$	0.26/0.78	0.80–100.0
Caffeic acid	N	-20	2.4493	-0.0938	0.9989	$1.85 \cdot 10^{-2}$	0.03/0.08	0.1–100.0
Ellagic acid	N	-30	0.9114	-0.6312	0.9887	$6.37 \cdot 10^{-2}$	0.23/0.70	0.7–100.0
Ellagic acid 3,3′-dimethyl ether	N	-35	0.6370	-0.4521	0.9872	$6.11 \cdot 10^{-2}$	0.32/0.96	1.0–100.0
Gallic acid	N	-10	2.6538	-0.1376	0.9990	$1.17 \cdot 10^{-2}$	0.01/0.04	0.10–100.0
Hyperoside = quercetin-3-*O*-β-D-galactopyranoside	N	-20	1.4689	-0.3641	0.9990	$5.69 \cdot 10^{-2}$	0.12/0.38	0.40–400.0
Isomyricitrin = myricetin 3-*O*-β-D-glucopyranoside	N	-20	2.6340	-0.2411	0.9973	$2.74 \cdot 10^{-2}$	0.03/0.10	0.10–350.0
Isoquercitrin = quercetin 3-*O*-β-D-glucopyranoside	N	-20	1.8267	-0.4160	0.9990	$11.73 \cdot 10^{-2}$	0.21/0.67	0.70–400.0
Juglanin = kaempferol 3-*O*- α-L-arabinopyranoside	N	-20	2.0384	-0.3640	0.9975	$2.02 \cdot 10^{-2}$	0.03/0.10	0.10–350.0
Kaempferol	N	-10	1.2416	-0.3615	0.9901	$3.02 \cdot 10^{-2}$	0.08/0.24	0.3–100.0
Kaempferol-3-*O*-β-D-glucuronopyranoside	N	-30	2.2126	-0.5160	0.9987	$8.11 \cdot 10^{-2}$	0.12/0.37	0.4–100.0
Miquelianin = quercetin-3-*O*-β-D-glucuronopyranoside	N	-20	1.6705	-0.4374	0.9988	$12.79 \cdot 10^{-2}$	0.25/0.77	0.8–100.0
Myricetin	N	-15	0.9375	-0.7631	0.9982	$1.42 \cdot 10^{-2}$	0.05/0.15	0.20–350.0
Myricetin-3-*O*-β-D-galactopyranoside	N	-20	2.4751	-0.2081	0.9976	$2.04 \cdot 10^{-2}$	0.03/0.10	0.10–350.0
Myricetin 3-*O*-rutinoside	N	-25	1.9634	-0.7458	0.9963	$2.59 \cdot 10^{-2}$	0.04/0.14	0.20–350.0
Myricitrin = myricetin-3-*O*-α-L-rhamnopyranoside	N	-20	1.8233	-0.7962	0.9975	$2.02 \cdot 10^{-2}$	0.04/0.11	0.20–350.0
Oenothein B	N	-35	0.8214	-0.2716	0.9893	$5.37 \cdot 10^{-2}$	0.22/0.65	0.7–100.0
Protocatechuic acid	N	-10	1.9610	-0.5271	0.9993	$0.94 \cdot 10^{-2}$	0.02/0.05	0.05–250.0
Quercetin	N	-15	1.1105	-0.3211	0.9937	$4.18 \cdot 10^{-2}$	0.12/0.38	0.40–400.0
Quercetin-3-*O*-(6′′-galloyl)-β-D-galactopyranoside	N	-25	1.1492	-0.6010	0.9980	$4.68 \cdot 10^{-2}$	0.14/0.41	0.50–400.0
Quercitrin = quercetin-3-*O*-α-L-rhamnopyranoside	N	-20	1.9871	-0.6871	0.9984	$5.63 \cdot 10^{-2}$	0.09/0.28	0.40–400.0
Rutin = quercetin-3-*O*-rutinoside	N	-20	1.2716	-0.7389	0.9897	$9.14 \cdot 10^{-2}$	0.23/0.72	0.80–400.0
Sinapic acid	N	-20	1.4238	-0.0891	0.9901	$7.33 \cdot 10^{-2}$	0.17/0.52	0.6–100.0

Table S2. *Cont.*

**Compound**	**Ionization** [a]	**CE** [b] **(eV)**	**Regression equation** [c]		r^2	S_{YX}	**LOD/ LOQ (µg/mL)**	**Linear range (µg/mL)**
			a	***b*·10**^{6}				
Tiliroside = kaempferol-3-*O*-(6′′-*O*-*p*-coumaroyl)-β-D-glucopyranoside	N	-30	2.3312	-0.4563	0.9803	14.92·10^{-2}	0.21/0.64	0.7–100.0

[a] Ionization mode : N—negative. [b] CE—collision energy. [c] Regression equation: $y = a \cdot x + b$.
